# Risk Stratification of Dengue Cases Requiring Hospitalization

**DOI:** 10.1002/jmv.70511

**Published:** 2025-07-24

**Authors:** Do Duc Anh, Mario Recker, Nguyen Trong The, Sanjeev Krishna, Peter G. Kremsner, Le Huu Song, Thirumalaisamy P. Velavan

**Affiliations:** ^1^ Institute of Tropical Medicine University of Tübingen Tübingen Germany; ^2^ Vietnamese German Center for Medical Research (VG‐CARE) Hanoi Vietnam; ^3^ Centre for Ecology and Conservation University of Exeter Penryn UK; ^4^ 108 Military Central Hospital Hanoi Vietnam; ^5^ School of Health and Medical Sciences, Institute of Infection and Immunity City St George's University of London London UK; ^6^ Centre de Recherches Médicales de Lambaréné (CERMEL) Lambarene Gabon; ^7^ Faculty of Medicine Duy Tan University Da Nang Vietnam

**Keywords:** biomarkers, clinical severity, dengue, inflammatory mediators, random forest

## Abstract

Dengue pathogenesis involves immune‐driven inflammation that contributes to severe disease progression. This study assessed a machine learning model to identify a minimal, yet highly predictive biomarker set, aiming to support clinical decision‐making and patient triage. A total of 48 inflammatory mediators were quantified from plasma samples collected at admission from confirmed dengue patients, classified as either dengue without warning signs (DF) or dengue with warning signs/severe dengue (DWS/SD). A random forest approach was applied to identify the most predictive biomarkers associated with disease severity requiring hospitalization, based on admission‐time variables. Among the 48 immune mediators, 43 were differentially expressed in dengue patients versus healthy controls, and 26 showed significant differences between DF and DWS/SD cases. Lymphocyte counts negatively correlated with IL‐1RA, while liver enzymes showed positive correlations with HGF and SCGF‐beta; platelet counts also negatively correlated with these markers. Key severity‐associated markers included HGF, TNF‐beta, MIP‐1‐beta, and SCGF‐beta. A model incorporating these markers and fever duration achieved nearly 80% accuracy in distinguishing DWS/SD from DF cases, independent of clinical examination. The findings suggest that targeted cytokine profiling may guide early hospitalization decisions and ease healthcare burdens in dengue‐endemic regions.

**Abbreviations**
Beta‐NGF: beta nerve growth factor
CTACK: cutaneous T‐cell attracting chemokine
Eotaxin: eotaxin (CCL11)
FGF‐basic: fibroblast growth factor basic
GM‐CSF: granulocyte macrophage colony stimulating factor
GRO‐alpha: growth regulated oncogene alpha
G‐CSF: granulocyte colony stimulating factor
HGF: hepatocyte growth factor
IFN‐alpha: interferon alpha
IFN‐gamma: interferon gamma
IL‐10: interleukin 10
IL‐12p40: interleukin 12 subunit p40
IL‐12p70: interleukin 12 subunit p70
IL‐13: interleukin 13
IL‐15: interleukin 15
IL‐16: interleukin 16
IL‐17: interleukin 17
IL‐18: interleukin 18
IL‐1RA: interleukin 1 receptor antagonist
IL‐1‐alpha: interleukin 1 alpha
IL‐1‐beta: interleukin 1 beta
IL‐2: interleukin 2
IL‐2R‐alpha: interleukin 2 receptor alpha
IL‐3: interleukin 3
IL‐4: interleukin 4
IL‐5: interleukin 5
IL‐6: interleukin 6
IL‐7: interleukin 7
IL‐8: interleukin 8
IL‐9: interleukin 9
IP10: interferon gamma‐induced protein 10
LIF: leukemia Inhibitory Factor
MCP1/MCAF: monocyte chemoattractant protein1/monocyte chemotactic and activating factor
MCP3: monocyte chemoattractant protein 3
MIF: macrophage migration inhibitory factor
MIG: monokine induced by gamma interferon
MIP1‐alpha: macrophage inflammatory protein 1 alpha
MIP1‐beta: macrophage inflammatory protein 1 beta
M‐CSF: macrophage colony stimulating factor
PDGF‐BB: platelet derived growth factor BB
RANTES: regulated on activation, normal t cell expressed and secreted
SCF: stem cell factor
SCGF‐beta: stem cell growth factor beta
SDF‐1‐alpha: stromal cell derived factor 1 alpha
TNF‐alpha: tumor necrosis factor alpha
TNF‐beta: tumor necrosis factor beta
TRAIL: tumor necrosis factor related apoptosis inducing ligand
VEGF: vascular endothelial growth factor

## 1 Introduction

Dengue is the most prevalent mosquito‐borne human disease and is caused by the dengue virus (DENV). Clinical manifestations range from asymptomatic to severe and sometimes fatal disease. Severe cases often require hospitalization where timely monitoring and intervention can reduce the overall mortality rate to less than 1% [1, 2]. The World Health Organization (WHO) classifies symptomatic dengue into three clinical categories: dengue without warning signs (DF; dengue fever), dengue with warning signs (DWS), and severe dengue (SD) [3]. Warning signs, such as mucosal bleeding, persistent vomiting, hepatomegaly, low platelet counts, and abruptly elevated liver enzymes, indicate the need to monitor patients closely, while milder cases can be treated at home [3]. Furthermore, cases with warning signs can eventually progress to SD leading to life‐threatening complications, such as hypovolaemic shock, severe bleeding, and multiorgan failure [4].

The symptoms of dengue are often difficult to differentiate from other viral fevers and concomitant illnesses, even during critical phases of infection [5]. This presents a significant challenge for clinicians attempting to provide an accurate diagnosis and prognosis of the disease, particularly in countries with a high prevalence of dengue. An inaccurate diagnosis may result in unnecessary hospitalization and subsequently an increased burden on the healthcare system [6]. On the other hand, the accurate identification of severe cases is critical to prevent fatalities. Clinical and conventional laboratory markers, including white blood cell (WBC) counts, platelet counts (PLT), and liver enzyme levels can help to distinguish severe cases of dengue from DF [7, 8]. However, clinical manifestations may be delayed and can either follow or be triggered by changes in humoral inflammatory profiles [9]. Therefore, inflammatory mediators such as cytokines and chemokines may serve as early direct indicators of the systemic inflammatory status and have potential for prognostication of tissue damage before clinical manifestations of disease severity [10].

Cytokines are fundamental to the pathogenesis and clinical manifestations of dengue. They are involved in both protection and pathogenesis by playing a crucial role in the control of DENV replication and the regulation of inflammatory processes. Dysregulated cytokine production causing excessive release of pro‐inflammatory cytokines may contribute to increased vascular permeability and increase disease severity and the risk of complications [11, 12]. Overproduction of Pro‐inflammatory cytokines such as tumor necrosis factor alpha (TNF‐alpha), interleukin (IL)‐1, IL‐6, and IL‐8, and the downregulation of anti‐inflammatory cytokines such as IL‐4, IL‐10, IL‐13, and tumor necrosis factor beta (TGF‐beta), can lead to systemic inflammation and vascular damage, worsening the dengue prognosis [12, 13].

This study aimed to develop a robust method for differentiating the severity of dengue infections, thereby facilitating rapid triage decisions based on a comprehensive assessment of humoral inflammatory markers. To achieve this, a broad spectrum of humoral immune mediators, including 48 cytokines, chemokines, and growth factors, was analyzed alongside clinical and laboratory parameters of dengue patients in Vietnam. A supervised machine learning approach was used to identify the most predictive biomarkers for determining disease severity requiring hospitalization, based on admission‐time variables. The analyses identified a small subset of cytokines capable of reliably distinguishing dengue severity, offering potential utility in early clinical decision‐making and patient triage.

## 2 Materials and Methods

### 2.1 Ethical Approval Statement

Signed informed consent was obtained from all study participants before enrollment. The study was approved by the Institutional Review Board of the 108 Military Hospital and the University of Tübingen for the project entitled “Host and viral factors influencing dengue severity and susceptibility” (ethics approval no. 274/2022B02). The study adhered to the Nagoya Protocol and received approval from the Vietnamese Ministry of Natural Resources and Environment (Reference: No.2995/QĐ‐BTNMT). All procedures followed GCP/GCLP guidelines.

### 2.2 Study Population

Samples were collected during two consecutive seasonal dengue outbreaks between September to December in 2021 and 2022. The study included civilian patients (*n* = 306) with symptoms of viral hemorrhagic fever, who were admitted to the 108 Military Central Hospital in Hanoi, Vietnam. Dengue was diagnosed according to the diagnostic criteria of the World Health Organisation (https://apps.who.int/iris/handle/10665/44188), which were adopted by the Vietnamese Ministry of Health. The inclusion criteria were patients presenting fever with at least two of the clinical sign/symptoms suggesting dengue (e.g., nausea/vomiting, headache, retro‐ocular pain, rash, body aches, Tourniquet test positive) and/or positive for at least one of the indirect diagnostic methods (serological rapid tests), as detailed in the WHO guideline 2009 [3]. Exclusion criteria included patients with bacterial or other viral infections, chronic diseases, or hematological disorders. Blood samples were collected from all dengue patients on admission. Similarly, blood samples were also collected from healthy blood donors (*n* = 118) who had tested negative for HBsAg, anti‐HCV and anti‐HIV from the hospital transfusion department. Plasma was separated from blood and stored at −70°C until use.

### 2.3 Dengue Serological Tests

Samples were subjected to nonstructural protein 1 (NS1) DENV antigen testing and anti‐DENV immunoglobulin M and G (IgM and IgG) antibody tests using the Bioline Dengue Duo kit (Abbott, Santa Clara, USA; formerly Alere Inc, Waltham, USA), following the manufacturer's instructions. Among dengue patients, those testing IgG positive within 8 days after the onset of fever were categorized as secondary infections, while cases testing positive only for NS1 or IgM were classified as primary infections. Tertiary and quaternary infections were indistinguishable from secondary infections in this study.

### 2.4 Dengue RNA Positivity and Exclusion of Zika/Chikungunya RNA

Total viral RNA was isolated from 140 µL of patient plasma utilizing the QIAmp Viral RNA Mini Kit (Qiagen GmbH, Hilden, Germany) in accordance with the manufacturer's guidelines. To exclude possible infections with other arboviruses circulating in Vietnam and to confirm dengue infection, all samples (*n* = 306) were subjected to multiplex real‐time PCR analysis for dengue/Zika/chikungunya viral RNA using the Fast Track Diagnostics Kit (Siemens Healthcare GmbH, Erlangen, Germany) on a LightCycler480‐II (Roche, Mannheim, Germany), following the manufacturer's protocol. Confirmed dengue cases (*n* = 299) were identified as those with detectable DENV RNA by real‐time RT‐PCR and tested negative for Zika/chikungunya RNA.

### 2.5 Clinical Severity and Laboratory Parameters

Patients were categorized clinically into three severity levels based on WHO guidelines [3]: dengue without warning signs (DF), DWS, and SD. The clinical presentation was recorded on admission, and the patient's sex (male or female) was noted as specified at birth. Laboratory parameters (see Table 1), including aspartate aminotransferase (AST) and alanine aminotransferase (ALT) levels, leukocytes (WBC) count, lymphocytes (LYM) count, neutrophils (NEU) count, Eosinophils (EOS) count, Basophils (BASO) count, erythrocytes (RBC) count, monocytes (MONO) count, platelet (PLT) count, hemoglobin (Hb), and hematocrit (HCT), were determined at admission to hospital.

**Table 1:** Patient characteristics on admission.

	Dengue without warning signs (DF) (*n* = 172)	Dengue with warning signs (DWS) and severe dengue (SD) (*n* = 127)	*p* value
Demographic data			
Median age (years)	47 (14–87)	49 (17–83)	0.233
Sex (% male)	54	50	0.64
Clinical presentations			
Days of fever (days)	4 (1–8)	5 (1–8)	< 0.001
Headache, *n* (%)	157 (91.3%)	112 (88.2%)	0.437
Retro ocular pain, *n* (%)	95 (55.2%)	92 (72.4%)	0.003
Myalgia, *n* (%)	129 (75.0%)	97 (76.4%)	0.781
Arthralgia, *n* (%)	112 (65.1%)	92 (72.4%)	0.198
Rash, *n* (%)	28 (16.3%)	73 (57.5%)	< 0.001
Vomit, *n* (%)	23 (13.4%)	33 (26.0%)	0.01
Abdominal pain, *n* (%)	0 (0%)	22 (17.3%)	NA
Lethargy, *n* (%)	0 (0%)	5 (3.9%)	NA
Hepatomegaly, *n* (%)	0 (0%)	3 (2.4%)	NA
Shock, *n* (%)	0 (0%)	5 (3.9%)	NA
Respiratory distress, *n* (%)	0 (0%)	6 (4.7%)	NA
Edema, *n* (%)	0 (0%)	31 (24.4%)	< 0.001
Bleeding manifestation			
Subcutaneous, *n* (%)	33 (19.2%)	86 (67.7%)	< 0.001
Mucosal, *n* (%)	0 (0%)	65 (51.2%)	< 0.001
Severe, *n* (%)	0 (0%)	4 (3.1%)	0.031
Laboratory tests			
Leukocytes ×10^3^/μL	4.01 (0.93–16.9)	3.71 (1.33–11.6)	0.947
Lymphocyte ×10^3^/μL	0.78 (0.17–4.57)	0.99 (0.22–4.09)	0.003
Neutrophils ×10^3^/μL	2.18 (0.45–203)	1.80 (0.74–7.70)	0.004
Eosinophils ×10^3^/μL	0.01 (0.01–0.24)	0.02 (0.01–0.45)	0.132
Basophils ×10^3^/μL	0.03 (0.01–1.18)	0.07 (0.02–1.76)	< 0.001
Monocytes ×10^3^/μL	0.36 (0.05–1.35)	0.31 (0.08–2.53)	0.140
Platelets ×10^3^/μL	115 (9.00–384)	20.0 (4.00–228)	< 0.001
Erythrocytes ×10^6^/μL	4.90 (3.66–7.62)	5.10 (2.76–6.81)	0.001
Hemoglobin g/dL	14.6 (11.0–18.7)	15.1 (10.0–19.0)	0.003
Hematocrit (%)	43.1 (31.8–54.7)	44.5 (21.4–60.5)	0.002
AST (U/L)	54.0 (15.1–1210)	119 (16.0–11 100)	< 0.001
ALT (U/L)	38.7 (8.00–855)	66.9 (8.20–2190)	< 0.001
Serological tests			
NS1—positivity (%)	127 (73.8%)	91 (71.7%)	0.695
IgM—positivity (%)	62 (36.0%)	79 (62.2%)	< 0.001
IgG—positivity (%)	56 (32.6%)	77 (60.6%)	< 0.001

*Note:* Variables were summarized in absolute count with percentage or median with (range). Categorical variables were compared using chi‐square test, and quantitative variables were compared using Wilcoxon test. *p*‐value < 0.05 is considered significant.

Abbreviations: AST, aspartate aminotransferase; ALT, alanine Aminotransferase; DENV, dengue virus; IgG, immunoglobulin G; IgM, immunoglobulin M; NS1, nonstructural protein.

### 2.6 Human Cytokines Screening Using Bio‐Plex Panel

Plasma samples from confirmed dengue cases by RT‐PCR (*n* = 299/306) and healthy controls (*n* = 118) were analyzed for 48 inflammatory markers, including cytokines (*n* = 35), chemokines (*n* = 9), and growth factors (*n* = 4). The assay was carried out using the magnetic bead‐based Bio‐Plex Pro Human Cytokine Screening Panel 48‐plex (Bio‐Rad Laboratories GmbH, Feldkirch, Germany) following the manufacturer's protocol on a Bio‐Plex 200 system.

The panel involves a biologically relevant array of adaptive immunity cytokines, pro‐inflammatory cytokines, and anti‐inflammatory cytokines, as: FGF‐basic, Eotaxin, G‐CSF, GM‐CSF, IFN‐gamma, IL‐1‐beta, IL‐1RA, IL‐1‐alpha, IL‐2R‐alpha, IL‐3, IL‐12p40, IL‐16, IL‐2, IL‐4, IL‐5, IL‐6, IL‐7, IL‐8, IL‐9, GRO‐alpha, HGF, IFN‐alpha, LIF, MCP3, IL‐10, IL‐12p70, IL‐13, IL‐15, IL‐17, IP10, MCP1/MCAF, MIG, Beta‐NGF, SCF, SCGF‐beta, SDF‐1 alpha, MIP1‐alpha, MIP1‐beta, PDGF‐BB, RANTES, TNF‐alpha, VEGF, CTACK, MIF, TRAIL, IL‐18, M‐CSF, and TNF‐beta (detailed in the abbreviations list).

Each assay utilized a 15 µL volume of plasma sample, with the assay controls, standards, and patient/healthy control samples distributed into each well of the assay 96‐well plate. All assay steps were conducted as per the manufacturer's instructions. The resulting data were processed using Bio‐Plex Manager 6.0 software (Bio‐Rad Laboratories, Hercules, CA, USA).

### 2.7 Statistical Analysis

The data were analyzed and visualized using R software version 4.3.2 (http://www.r-project.org). A *p*‐value < 0.05 was considered statistically significant for statistical comparisons in the study. Demographic, clinical, and laboratory data of the patients were summarized, with quantitative variables presented as median values with ranges, and categorical variables as absolute numbers with percentages. The titers of quantified biomarkers were expressed as median values with ranges (pg/mL). The distribution of quantitative variables was evaluated for normality using the Shapiro–Wilk and D'Agostino–Pearson tests, with additional visual assessment based on skewness and kurtosis. Categorical variables were compared using chi‐square tests, while continuous variables were assessed using the Wilcoxon rank‐sum test. Nonparametric Spearman correlation with Holm corrections for multiple testing was utilized to assess correlations between different markers and laboratory parameters.

### 2.8 Predictive Modelling

A random forest machine learning approach [14], using the “r*andomForest*” package in R [15], was taken to identify the most important variables for differentiating dengue cases requiring close medical observation (DWS/SD) from milder cases (DF) [3]. For this, the study considered two different sets of predictors. The first was based solely on the plasma levels inflammatory mediators together with the duration of fever before hospital admission (days of fever). The second was based on more traditional dengue severity‐related parameters that included age, sex, days of fever, type of infection (primary/secondary dengue), clinical features, and laboratory parameters (as listed in Table 1). Warning signs, such as mucosa/severe bleeding manifestations, abdominal pain, lethargy, hepatomegaly, shock, and respiratory distress were excluded due to DWS/SD being partially defined based on the presence of these symptoms. Seven inflammatory biomarkers (GRO‐alpha, beta‐NGF, IL‐15, IL‐3, IL‐5, VEGF and IL‐6) with a missing rate of >30% were excluded from the analysis (Table 2); other missing values were imputed using the “*missForest*” package in R [16]. Feature selection was carried out using the “*VSURF*’ R package” [17]. Model metrics, including accuracy, specificity, sensitivity, and F1‐score, were generalized following 10‐fold cross‐validation. The receiver operating characteristic curve was constructed and the area under the curve (AUROC) was calculated using the “*pROC*” package in R [18].

**Table 2:** Plasma levels of inflammatory mediators in dengue patients.

	Dengue without warning signs (DF) (*n* = 172)	Dengue with warning signs (DWS) and severe dengue (SD) (*n* = 127)	*p* value
Pro‐inflammatory			
IL‐17	8.7 (1.2–49)	11 (1.6–36)	0.001
MIG	1084 (61–51 615)	2140 (63–60 703)	0.010
MIP1‐alpha	3.9 (0.28–36)	5.9 (0.81–64)	0.010
SCGF‐beta	119 349 (5887–1 806 133)	384 276 (9043–1 093 594)	< 0.001
GRO‐alpha*	766 (92–2500)	399 (54–2242)	< 0.001
IFN‐gamma	40 (0.87–346)	32 (0.61–269)	0.005
MCP1/MCAF	81 (4.2–1505)	57 (6.1–977)	< 0.001
Beta‐NGF*	4.1 (0.06–86)	3.7 (0.03–67)	0.390
IL‐15*	374 (23–774)	101 (14–284)	0.009
IL‐18	70 (2.1–947)	92 (6–557)	0.091
IP10	10 234 (80–615 856)	13 824 (333–388 336)	0.641
M‐CSF	49 (3.9–298)	60 (2.5–343)	0.415
MCP3	4 (0.12–194)	4.1 (0.38–193)	0.802
TNF‐alpha	58 (6.4–361)	60 (11–454)	0.539
TRAIL	79 (2.5–558)	117 (1.2–444)	0.365
Eotaxin	91 (7.7–960)	131 (13–480)	0.045
IL‐3*	0.4 (0.04–9)	0.68 (0.02–51)	0.058
MIF	444 (19–5570)	494 (27–15 730)	0.88
PDGF‐BB	355 (1–6773)	326 (3.6–4513)	0.458
MIP1‐beta	140 (7.6–783)	68 (6.4–283)	< 0.001
RANTES	1129 (63–59 856)	484 (29–14 771)	< 0.001
TNF‐beta	146 (11–404)	64 (2.4–357)	< 0.001
GM‐CSF	2.4 (0.03–12)	2.2 (0.03–11)	0.4
IL‐5*	53 (4.5–278)	23 (7–112)	0.008
SDF‐1‐alpha	1920 (218–14 677)	1585 (268–16 000)	0.295
Anti‐inflammatory			
CTACK	950 (27–9892)	1682 (68–6116)	0.003
IL‐10	9.9 (0.07–461)	19 (0.03–183)	< 0.001
IL‐1RA	1231 (66–20 578)	756 (82–26 298)	< 0.001
IL‐4	2.3 (0.25–13)	2.2 (0.33–5.7)	0.608
IL‐9	185 (20–562)	89 (5.8–523)	< 0.001
IL‐7	9.6 (0.18–108)	8.3 (0.18–107)	0.12
Pro/anti‐inflammatory			
HGF	600 (64–3059)	1294 (86–12 358)	< 0.001
IL‐1‐alpha	27 (0.12–205)	53 (2.2–342)	< 0.001
IL‐1‐beta	6.6 (0.31–34)	7.8 (1.4–20)	0.032
IL‐12p40	77 (0.85–456)	95 (6.9–524)	0.015
IL‐13	5 (0.12–61)	7 (0.15–40)	0.002
LIF	44 (3.8–327)	82 (3.8–315)	0.003
IL‐2R‐alpha	77 (4.5–689)	97 (6.5–472)	0.063
IFN‐alpha	13 (0.08–104)	11 (0.63–293)	0.169
IL‐6*	4.9 (0.03–360)	4.7 (0.09–502)	0.774
IL‐12p70	2.8 (0.07–39)	3.8 (0.07–40)	0.239
IL‐16	83 (5.4–1272)	104 (9.9–2236)	0.104
IL‐2	6.4 (0.04–50)	5.1 (0.1–23)	0.26
IL‐8	16 (0.9–181)	24 (2.9–790)	0.002
Growth factors			
FGF‐basic	36 (0.46–234)	66 (1.8–291)	0.002
G‐CSF	131 (8.8–937)	136 (12–8649)	0.333
SCF	83 (8.3–287)	94 (11–885)	0.203
VEGF*	299 (10–1010)	93 (21–876)	0.006

*Note:* Variables were summarized in median with range (pg/mL). The names of 48 inflammatory mediators are detailed in the abbreviation list. *Variables with > 30% values missing from measurement. Levels of variables were compared using Wilcoxon test. *p*‐value < 0.05 is considered significant.

## 3 Results

### 3.1 Confirmation of Dengue Infection and Classification of Primary and Secondary Dengue

Zika and chikungunya viral RNA were not detected in any of the 306 tested samples. The multiplex real‐time RT‐PCR assay confirmed the presence of DENV RNA in 299 out of 306 cases; the other seven cases were excluded from the study. IgM/IgG positivity was used to differentiate primary dengue infections (133/299) from secondary infections (140/299). Among DENV RT‐PCR confirmed cases included in the study, 26 cases (*n* = 26/299) with negative dengue serological tests (negative for NS1, IgG, and IgM assays) were retained in the analysis due to their positive RT‐PCR results, although they could not be classified as either primary or secondary infections.

### 3.2 Baseline Characteristics of the Study Participants

Both patients and healthy controls resided in Hanoi metropolitan areas and were of Kinh ethnicity. No significant differences were observed in age or sex distribution between controls and patients, nor between the different dengue severity groups (median age, DF vs. DWS/SD: 47 vs. 49 years, *p* = 0.233; proportion male, DF vs. DWS/SD: 54% vs. 50%, *p* = 0.64) (Table 1). Patients were categorized into two groups based on the need for hospitalization: those with dengue without warning signs (DF, *n* = 172), and those with DWS or SD (DWS/SD, *n* = 127; including *n* = 114 with DWS and *n* = 13 with SD).

The clinical data of the patients are summarized in Table 1. Significant variations were observed in the duration of fever before admission (days of fever), dengue‐related clinical manifestations, blood parameters, and liver enzymes between the DF and DWS/SD groups (Table 1). Retro‐ocular pain (*p* = 0.003), rash (*p* < 0.001), vomiting (*p* = 0.01), and bleeding manifestations (*p* < 0.001) were more frequently reported in DWS/SD compared to DF patients (Table 1). DWS/SD patients exhibited significantly lower PLTs (*p* < 0.001) and higher levels of liver enzymes AST (*p* < 0.001), and ALT (*p* < 0.001) compared to DF patients (Table 1).

### 3.3 Level of Inflammatory Mediators Differ Between Dengue Patients and Healthy Controls

Plasma concentrations of *n* = 43/48 markers were found to be significantly different between dengue patients and healthy controls, except for *n* = 5/48 markers including TRAIL (*p* = 0.082), TNF‐beta (*p* = 0.991), GM‐CSF (*p* = 0.405), IL‐5 (*p* = 0.078), and IL‐7 (*p* = 0.051) (Supporting Information S1: Table S1). In addition, significant differences between DF patients and DWS/SD patients were observed in *n* = 26/48 markers (Table 2), including Pro‐inflammatory markers (*n* = 13): IL‐17, MIG, MIP1‐alpha, SCGF‐beta, GRO‐alpha, IFN‐gamma, MCP1/MCAF, IL‐15, Eotaxin, MIP1‐beta, RANTES, TNF‐beta, IL‐5; anti‐inflammatory markers (*n* = 4): CTACK, IL‐10, IL‐1RA, IL‐9; Pro/anti‐inflammatory markers (*n* = 7): HGF, IL‐1‐alpha, IL‐1‐beta, IL‐12p40, IL‐13, LIF, IL‐8; and two growth factors (*n* = 2): FGF‐basic, VEGF (Table 2).

### 3.4 Correlations of Inflammatory Mediators and Laboratory Parameters in Dengue

**Figure 1:**
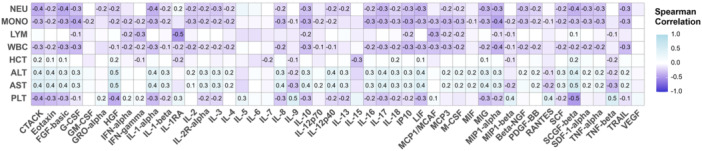
Correlations between laboratory parameters and inflammatory biomarkers. The names of inflammatory mediators are detailed in the abbreviation list. The correlation coefficient was calculated using Spearman's method with Holm corrections. *p*‐value < 0.05 is considered significant. ALT, alanine aminotransferase; AST, aspartate aminotransferase; HCT, haematocrit; LYM, lymphocytes count; MONO, monocytes count; NEU, neutrophils count; PLT, platelet count; WBC, leucocytes count.

Spearman correlations were employed to determine the relationship between the plasma levels of 48 inflammatory mediators and conventional laboratory parameters in dengue patients, as summarized in Figure 1 and Supporting Information S1: Table S2. Notably, a negative correlation was observed between lymphocyte counts and IL‐1RA (*ρ* = –0.5, *p* < 0.001), while positive correlations were noted between liver enzymes and HGF (AST: *ρ* = 0.5, *p* < 0.001; ALT: *ρ* = 0.5, *p* < 0.001) and SCGF‐beta (AST: *ρ* = 0.5, *p* < 0.001; ALT: *ρ* = 0.4, *p* < 0.001) (Figure 1). PLTs were also observed to negatively correlated with HGF (*ρ* = –0.4, *p* < 0.001) and SCGF‐beta (*ρ* = –0.5, *p* < 0.001), suggesting the potential associations of HGF and SCGF‐beta with dengue (Figure 1 and Supporting Information S1: Table S2).

**Figure 2:**
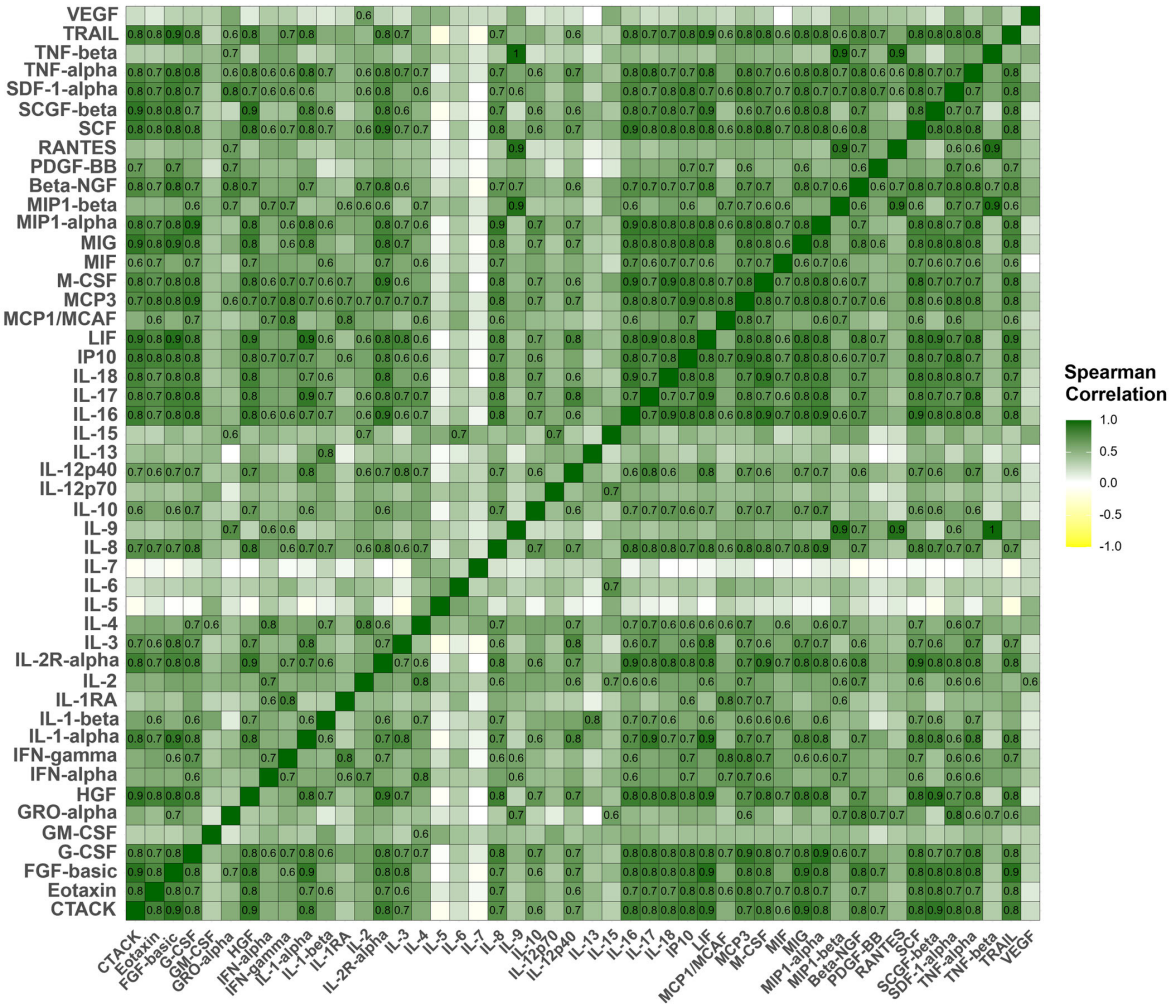
Correlations among inflammatory biomarkers. The names of inflammatory mediators are detailed in the abbreviation list. The correlation coefficient was calculated using Spearman's method with Holm corrections. *p*‐value < 0.05 is considered significant.

In addition, other blood cell counts (WBC, NEU, and MONO) displayed inverse correlations with majority of markers, except for IL‐5, IL‐6, IL‐7, IL‐15, TNF‐beta, and VEGF (Figure 1). Correlations among 48 inflammatory mediators were also summarized in Figure 2 and Supporting Information S1: Table S3. While strong correlations were found between majority of mediators, lower correlations with other cytokines were observed in IL‐5, IL‐6, and IL‐7 (Figure 2). These results indicate correlations among all variables analyzed in the study, including inflammatory biomarkers and conventional dengue parameters, suggesting the presence of multicollinearity.

### 3.5 Cytokine‐Based Classification of Dengue Severity

**Figure 3:**
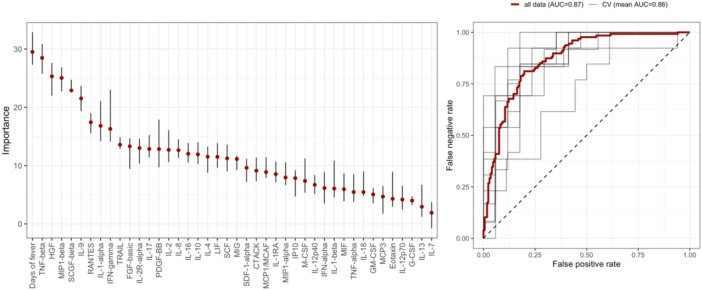
Feature importance plot and AUROC curve of Model 1. The names of inflammatory mediators are detailed in the abbreviation list. AUC, area under the curve; CV, cross‐validation.

**Figure 4:**
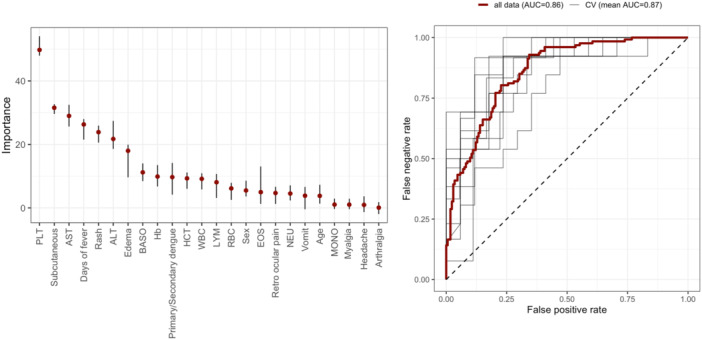
Feature importance plot and AUROC curve of Model 2. ALT, alanine aminotransferase; AST, aspartate aminotransferase; AUC, area under the curve; BASO, basophils count; CV, cross‐validation; EOS, eosinophils count; Hb, haemoglobin; HCT, haematocrit; LYM, lymphocytes count; MONO, monocytes count; NEU, neutrophils count; PLT, platelet count; RBC, erythrocytes count; WBC, leucocytes count.

The ability of inflammatory mediators to differentiate patients by dengue severity, and thus their need for hospitalization, was subsequently investigated. Following a feature selection procedure (see Section 2), four cytokines (TNF‐beta, HGF, MIP1‐beta, and SCGF‐beta), alongside the days of fever, were identified as robust predictors for disease severity. A model based on these features (Model 1) showed a predictive accuracy in differentiating DWS/SD from DF of 0.78 and an AUROC of 0.86 (Figure 3 and Table 3), with model sensitivity and specificity of 0.81 and 0.77, respectively. Importantly, model performance based on these features alone was comparable to a model based on traditional dengue markers including clinical and laboratory parameters (Model 2) (accuracy = 0.77, AUROC = 0.87, sensitivity = 0.78, specificity = 0.78) (Figure 4 and Table 3), implying that these cytokines could be used as robust biomarkers of dengue severity for rapid patient triaging. “A reduced model derived from Model 2, incorporating the strongest predictors, including days of fever, TNF‐beta, HGF, MIP1‐beta, and SCGF‐beta achieved comparable, and slightly improved, performance relative to the full Model 2 (accuracy = 0.78, AUROC = 0.88, sensitivity = 0.79, specificity = 0.76) (Supporting Information S1: Figure S1).”

**Table 3:** Parametric of Random Forest models after 10‐fold cross‐validation.

	Accuracy	AUROC	Sensitivity	Specificity	F1‐score
Model 1	0.78	0.86	0.81	0.77	0.81
Model 2	0.77	0.87	0.78	0.78	0.79

## 4 Discussion

In dengue, patient triaging remains challenging due to the nonspecific signs and symptoms of the disease. Conventional classification of dengue cases relies on astute clinical interpretation and laboratory findings [5]. This approach requires experienced physicians and may not be optimal during dengue outbreaks. Therefore, this study aimed to determine potential biomarkers that could assist physicians in making timely admission decisions for dengue patients based on inflammatory biomarkers, independently of clinical symptoms.

The study revealed significant differences in plasma levels of inflammatory mediators between dengue patients and healthy controls, as well as between DF and DWS/SD patients. A total of 43 out of 48 markers were differentially regulated between dengue patients and healthy controls, highlighting the altered humoral inflammatory profile associated with dengue infection. Markers that did not show significant differences between patients and controls suggest that these distinct cytokines may not play a prominent role in dengue‐associated immune dysregulation, or that their levels remain stable across different stages of the disease.

Significant differences were found in 26 of 48 markers between DF and DWS/SD dengue patients. Among the pro‐inflammatory markers, levels of IFN‐γ, MIP‐1β, RANTES, and TNF‐β were significantly higher in DF patients compared to those with DWS/SD, whereas SCGF‐β levels were significantly elevated in the DWS/SD group (Table 2). These markers play key roles in immune cell activation, chemotaxis, and the inflammatory cascade, processes typically upregulated during the acute phase of dengue infection [12, 19]. Strong associations with severity were also found in anti‐inflammatory markers, including IL‐10, IL‐1RA and IL‐9, suggesting possible disruptions of the balance between pro‐inflammatory and anti‐inflammatory responses in cases that required hospitalization. Additionally, inflammatory markers with both pro‐inflammatory and anti‐inflammatory effects, such as HGF, IL‐1‐alpha, and IL‐1‐beta, were significantly elevated in DWS/SD patients. This may reflect the complex interplay between inflammation and immune resolution during disease progression [11]. Our results indicate that FGF‐basic levels are elevated in DWS/SD patients, while VEGF levels are reduced, highlighting a potential imbalance that may impair effective tissue repair and contribute to increased vascular permeability during infection. Elevated FGF‐basic levels in DWS/SD patients may reflect a compensatory response to greater endothelial damage or tissue injury in more severe disease, triggering enhanced fibroblast activation and repair mechanisms [20]. Notably, these observations contrast with findings by Furuta et al., who reported reductions in both FGF‐basic and VEGF levels in DHF patients compared to DF patients, suggesting that differences in timing, disease kinetics, or host response dynamics may influence growth factor profiles [21]. Further studies with longitudinal data are needed to elucidate the temporal dynamics and regulatory roles of these growth factors in dengue pathogenesis.

With several inflammatory mediators as potential markers for distinguishing DWS/SD from DF cases, a machine learning approach was applied. After a robust feature selection process, the final model comprises five variables: days of fever, TNF‐beta, HGF, MIP‐1‐beta, and SCGF‐beta, which can differentiate DWS/SD from DF cases with nearly 80% accuracy. For years, conventional laboratory parameters such as PLT, HCT, and liver enzymes have been used as indicators of disease severity, alongside clinical manifestations [8, 22]. A comprehensive clinical examination provides substantial data, whereas a less thorough approach may compromise the specificity and sensitivity of outcome prediction [22, 23]. Therefore, the model's independence from clinical examination in this study is a key advantage, making it particularly useful when clinical presentation is unclear and patient numbers are high during an outbreak [5].

The time from symptom onset plays a crucial role in determining the severity of dengue. This variable is often measured from the onset of initial symptoms, most commonly fever, which is both easily recognized and widely reported by patients. A recent study has also shown that the time since dengue symptom onset is one of the most important predictors for the length of hospital stays, independent of the assigned severity score [7]. This variable is closely linked to viraemia levels, which significantly impact disease progression [24]. In addition, the disease time‐point is strongly associated with dynamic humoral changes, including fluctuations in inflammatory mediators and immune responses that influence dengue severity [19]. The study analyses thus reconfirm the importance of considering the number of days of fever alongside other dengue‐related variables.

During its lifecycle, DENV infects hepatocytes and Kupffer cells, resulting in liver damage and further exacerbation of liver dysfunction [25]. HGF, a protein produced in response to liver injury, is elevated in SD patients and correlates with liver enzyme levels. Consistent with previous studies [26], HGF was also identified as a potential predictor of DWS/SD. Meta‐analyses have similarly shown that the initial acute inflammatory response with hepatic involvement is a key determinant of disease progression in dengue [27], suggesting the promising value of HGF for dengue severity prediction.

In contrast to HGF, TNF‐beta levels were significantly higher in patients with DF (milder cases) compared to those with DWS/SD, indicating an association between elevated TNF‐beta and less severe clinical presentations. Since TNF‐beta is known to inhibit viral replication and synergize with other interferons [28], reduced TNF‐beta levels may favor DENV activity and increase host damage. The results also demonstrated that TNF‐beta plasma levels were significantly higher in healthy controls than in dengue patients, suggesting a potential protective role for this cytokine against dengue infection and progression to severe disease. In addition to the possible protective role of TNF‐beta, MIP1‐beta showed a strong association with DF patients. Consistent with the finding from this study, Bozza et al. identified MIP1‐beta as a predictive factor with a protective effect for SD [29].

During dengue progression, there is a notable decrease in leukocyte count [29], which is counteracted by increased haematopoiesis following the upregulation of bone marrow‐stimulating factors, such as SCGF‐beta [30]. Previous reports have indicated an increased secretion of SCGF‐beta following infection with respiratory syncytial virus [31] and in cases of liver cancer [32]. This study revealed significant correlations between SCGF‐beta levels and monocyte, leukocyte, and PLTs, as well as AST and ALT levels, suggesting an upregulation of hematopoietic activity to compensate for blood and other tissue damage.

Changes in laboratory parameters reflect the systemic impact of dengue, with thrombocytopenia and transaminitis as key indicators of disease severity. It is thus rational to find strong correlations between most inflammatory mediators and dengue‐related laboratory parameters, which also aligns with previous studies that have shown how these factors collectively contribute to immune responses and disease pathogenesis [11, 27]. Furthermore, the findings from this study proposed that inflammatory markers could serve as early indicators of disease progression, potentially before these could be detected through laboratory‐assessed abnormalities, such as thrombocytopenia or elevated liver enzymes [9]. Moreover, and as clearly demonstrated here, a small number of measured cytokines are sufficient to identity patients in need of close medical observation as reliable as traditional assessment based on time‐consuming laboratory findings.

The complexity of dengue pathogenesis arises from the interaction of various factors, including viral and host elements, with the host immune system playing a pivotal role. This study demonstrates that among other biomarkers, TNF‐beta, HGF, MIP‐1 beta, and SCGF‐beta are strongly associated with dengue, and together with duration of symptoms before hospital admission, these markers could help identify cases requiring hospitalization on admission. Nonetheless, a comprehensive clinical examination and case‐by‐case decision‐making remain vital in medical practice. It is also important to note that the classification of DF and DWS/SD in this study was based on patients' clinical evaluations, and the possibility of misclassification by attending physicians cannot be excluded. Therefore, a longitudinal study with severity assessments at different time points may provide further insights into the role of various humoral mediators in dengue. One additional limitation is that this was a single center study conducted in Vietnam. Therefore, the generalizability of these findings to other geographic regions and dengue serotypes requires further investigation. Nonetheless, this study was able to identify distinct humoral inflammatory profiles corresponding to different levels of dengue severity and proposed potential biomarkers as indicators for patient admission. While the large panel of inflammatory markers examined in this study provided comprehensive insights into dengue immunopathogenesis, it was intentionally applied to cast the net wide and identify a small set of robust predictors of severity. Routine measurement of all analytes is not proposed; instead, future efforts should focus on validating a minimal, cost‐effective biomarker subset with comparable predictive power for clinical application. As dengue outbreaks progress rapidly and strain healthcare systems, this approach is promising to reduce the burden on medical facilities in endemic areas.

## 5 Conclusion

Key inflammatory biomarkers and the duration of symptoms before admission can together inform hospitalization decisions in dengue patients. Targeted cytokine profiling integrated with machine learning offers a practical approach to improve triage and reduce healthcare burden in dengue‐endemic areas. Further longitudinal studies are needed to validate these findings.

## Author Contributions

T.P.V. and L.H.S. conceptualized and designed the study. L.H.S., P.G.K., and T.P.V. contributed to the study materials and assays. N.T.T recruited the patients. D.D.A. performed the experimental procedures. D.D.A., M.R., and S.K. were involved in the statistical analysis and validation of the results. T.P.V., D.D.A., and M.R. wrote the first draft. All authors have read and approved the manuscript.

## Conflicts of Interest

The authors declare no conflicts of interest.

## Supporting information


**Supplementary Table S1.** Plasma levels of inflammatory mediators in dengue patients and healthy controls.
**Supplementary Table S2.** Correlation matrix of plasma levels of inflammatory mediators and laboratory parameters in dengue patients.
**Supplementary Table S3.** Correlation matrix of plasma levels of inflammatory mediators in dengue patients.
**Supplementary Figure S1.** Feature importance plot and AUROC curve of Model with the most robust predictors.

## Data Availability

The data that supports the findings of this study are available in the supporting information of this article.
