# Supplementary material for: Risk Stratification of Dengue Cases Requiring Hospitalization
Source: J Med Virol. 2025 Jul 24;97(8):e70511. doi: 10.1002/jmv.70511 (PMC12288109; doi:10.1002/jmv.70511)

**Table of Contents**

[**Abbreviations List** 2](#_Toc202532871)

[**Supplementary Table S1.** Plasma levels of inflammatory mediators in dengue patients and healthy controls 3](#_Toc202532872)

[**Supplementary Table S2.** Correlation matrix of plasma levels of inflammatory mediators and laboratory parameters in dengue patients 4](#_Toc202532873)

[**Supplementary Table S3.** Correlation matrix of plasma levels of inflammatory mediators in dengue patients 6](#_Toc202532874)

[**Supplementary Figure S1.** Feature importance plot and AUROC curve of Model with the most robust predictors 15](#_Toc202532875)

# **Abbreviations List**

FGF-basic: Fibroblast Growth Factor basic

Eotaxin: Eotaxin (CCL11)

G-CSF: Granulocyte Colony Stimulating Factor

GM-CSF: Granulocyte Macrophage Colony Stimulating Factor

IFN-gamma: Interferon gamma

IL-1-beta: Interleukin 1 beta

IL-1RA: Interleukin 1 receptor antagonist

IL-1-alpha: Interleukin 1 alpha

IL-2R-alpha: Interleukin 2 receptor alpha

IL-3: Interleukin 3

IL-12p40: Interleukin 12 subunit p40

IL-16: Interleukin 16

IL-2: Interleukin 2

IL-4: Interleukin 4

IL-5: Interleukin 5

IL-6: Interleukin 6

IL-7: Interleukin 7

IL-8: Interleukin 8

IL-9: Interleukin 9

GRO-alpha: Growth regulated oncogene alpha

HGF: Hepatocyte Growth Factor

IFN-alpha: Interferon alpha

LIF: Leukaemia Inhibitory Factor

MCP3: Monocyte Chemoattractant Protein 3

IL-10: Interleukin 10

IL-12p70: Interleukin 12 subunit p70

IL-13: Interleukin 13

IL-15: Interleukin 15

IL-17: Interleukin 17

IP10: Interferon gamma-induced protein 10

MCP1/MCAF: Monocyte Chemoattractant Protein1/Monocyte Chemotactic and Activating Factor

MIG: Monokine Induced by Gamma Interferon

Beta-NGF: Beta Nerve Growth Factor

SCF: Stem Cell Factor

SCGF-beta: Stem Cell Growth Factor beta

SDF-1-alpha: Stromal Cell Derived Factor 1 alpha

MIP1-alpha: Macrophage Inflammatory Protein 1 alpha

MIP1-beta: Macrophage Inflammatory Protein 1 beta

PDGF-BB: Platelet Derived Growth Factor BB

RANTES: Regulated on Activation, Normal T Cell Expressed and Secreted

TNF-alpha: Tumour Necrosis Factor alpha

VEGF: Vascular Endothelial Growth Factor

CTACK: Cutaneous T-Cell Attracting Chemokine

MIF: Macrophage Migration Inhibitory Factor

TRAIL: Tumour Necrosis Factor Related Apoptosis Inducing Ligand

IL-18: Interleukin 18

M-CSF: Macrophage Colony Stimulating Factor

TNF-beta : Tumor Necrosis Factor beta

# **Supplementary Table S1. Plasma levels of inflammatory mediators in dengue patients and healthy controls**

|  | **Healthy control**  **(n=118)** | **Patients (n=299)** | **p-value** |
| --- | --- | --- | --- |
| **Pro-inflammatory** |  | | |
| IL-17 | 3.8 [0.62 - 6.6] | 9.82 [1.19, 49.0] | < 0.001 |
| MIG | 149 [38 - 1239] | 1610 [61.0, 60700] | 0.044 |
| MIP1-alpha | 3.1 [0.6 - 45] | 4.92 [0.280, 64.3] | < 0.001 |
| SCGF-beta | 59300 [25181 - 151255] | 245000 [5890, 1810000] | < 0.001 |
| GRO-alpha | 304 [4.1 - 789] | 660 [53.8, 2500] | < 0.001 |
| IFN-gamma | 15 [5.2 - 53] | 36.6 [0.610, 346] | < 0.001 |
| MCP1/MCAF | 32 [7.9 - 211] | 67.4 [4.17, 1500] | < 0.001 |
| Beta-NGF | 0.92 [0.08 - 9.8] | 3.96 [0.0300, 85.8] | < 0.001 |
| IL-15 | 99 [9.4 - 363] | 311 [14.5, 774] | < 0.001 |
| IL-18 | 28 [4.7 - 244] | 79.0 [2.06, 947] | < 0.001 |
| IP10 | 463 [95 - 1608] | 12400 [79.8, 616000] | < 0.001 |
| M-CSF | 3.9 [0.44 - 30] | 55.0 [2.50, 343] | < 0.001 |
| MCP3 | 2.5 [0.06 - 33] | 4.08 [0.120, 194] | < 0.001 |
| TNF-alpha | 26 [15 - 59] | 59.7 [6.40, 454] | < 0.001 |
| TRAIL | 13 [5 - 37] | 93.6 [1.22, 558] | 0.082 |
| Eotaxin | 136 [14 - 504] | 104 [7.73, 960] | < 0.001 |
| IL-3 | 1.5 [0.02 - 5.3] | 0.510 [0.0200, 50.6] | < 0.001 |
| MIF | 4735 [647 - 145091] | 465 [18.8, 15700] | < 0.001 |
| PDGF-BB | 3444 [22 - 11044] | 336 [1.00, 6770] | < 0.001 |
| MIP1-beta | 252 [132 - 614] | 88.6 [6.42, 783] | < 0.001 |
| RANTES | 23896 [1874 - 154240] | 790 [29.0, 59900] | < 0.001 |
| TNF-beta | 309 [120 - 511] | 86.4 [2.44, 404] | 0.991 |
| GM-CSF | 2.2 [0.16 - 15] | 2.28 [0.0300, 12.0] | 0.405 |
| IL-5 | 32 [3 - 98] | 46.0 [4.53, 278] | 0.078 |
| SDF-1-alpha | 1932 [925 - 2774] | 1750 [218, 16000] | < 0.001 |
| **Anti-inflammatory** |  | | |
| CTACK | 388 [107 - 1219] | 1200 [26.9, 9890] | < 0.001 |
| IL-10 | 1.8 [0.6 - 19] | 13.5 [0.0300, 461] | < 0.001 |
| IL-1RA | 156 [37 - 1749] | 1000 [66.4, 26300] | < 0.001 |
| IL-4 | 1.2 [0.28 - 2] | 2.19 [0.250, 12.8] | < 0.001 |
| IL-9 | 288 [137 - 461] | 113 [5.80, 562] | < 0.001 |
| IL-7 | 6.7 [0.16 - 161] | 9.00 [0.180, 108] | 0.051 |
| **Pro/anti inflammatory** |  | | |
| HGF | 248 [94 - 731] | 745 [63.6, 12400] | < 0.001 |
| IL-1-alpha | 8 [0.44 - 60] | 40.3 [0.120, 342] | < 0.001 |
| IL-1-beta | 2 [0.42 - 30] | 7.02 [0.310, 33.6] | < 0.001 |
| IL-12p40 | 20 [12 - 138] | 87.0 [0.850, 524] | < 0.001 |
| IL-13 | 2.2 [0.34 - 21] | 5.52 [0.120, 61.2] | < 0.001 |
| LIF | 16 [2.1 - 81] | 63.8 [3.82, 327] | < 0.001 |
| IL-2R-alpha | 22 [3.3 - 133] | 83.7 [4.54, 689] | < 0.001 |
| IFN-alpha | 4.6 [0.04 - 8.4] | 11.8 [0.0800, 293] | < 0.001 |
| IL-6 | 0.78 [0 - 7.9] | 4.80 [0.0300, 502] | < 0.001 |
| IL-12p70 | 1.5 [0.04 - 16] | 3.17 [0.0700, 39.8] | < 0.001 |
| IL-16 | 106 [11 - 1147] | 93.3 [5.39, 2240] | 0.019 |
| IL-2 | 4 [1.4 - 8] | 5.48 [0.0400, 50.2] | < 0.001 |
| IL-8 | 46 [3.2 - 1997] | 18.1 [0.900, 790] | < 0.001 |
| **Growth factors** |  | | |
| FGF-basic | 15 [2.3 - 21] | 52.7 [0.460, 291] | < 0.001 |
| G-CSF | 31 [0.04 - 228] | 133 [8.78, 8650] | < 0.001 |
| SCF | 23 [7.5 - 100] | 87.8 [8.30, 885] | < 0.001 |
| VEGF | 86 [1.3 - 449] | 243 [9.96, 1010] | < 0.001 |

Variables were summarized in median with range (pg/mL). The names of inflammatory mediators are detailed in the abbreviation list. Levels of variables were compared using Wilcoxon test. P-value <0.05 is considered significant.

# **Supplementary Table S2. Correlation matrix of plasma levels of inflammatory mediators and laboratory parameters in dengue patients**

|  | **PLT** | **AST** | **ALT** | **HCT** | **WBC** | **LYM** | **MONO** | **NEU** |
| --- | --- | --- | --- | --- | --- | --- | --- | --- |
| **CTACK** | -0.354 | 0.423 | 0.376 | 0.191 | -0.264 | 0.072 | -0.296 | -0.389 |
| *p-value* | <0.001 | <0.001 | <0.001 | 0.001 | <0.001 | 0.234 | <0.001 | <0.001 |
| **Eotaxin** | -0.312 | 0.397 | 0.365 | 0.121 | -0.193 | -0.004 | -0.217 | -0.244 |
| *p-value* | <0.001 | <0.001 | <0.001 | 0.036 | 0.001 | 0.942 | <0.001 | <0.001 |
| **FGF-basic** | -0.281 | 0.355 | 0.303 | 0.144 | -0.319 | 0.019 | -0.332 | -0.397 |
| *p-value* | <0.001 | <0.001 | <0.001 | 0.013 | <0.001 | 0.761 | <0.001 | <0.001 |
| **G-CSF** | -0.12 | 0.262 | 0.258 | 0.004 | -0.318 | -0.15 | -0.393 | -0.296 |
| *p-value* | 0.038 | <0.001 | <0.001 | 0.945 | <0.001 | 0.013 | <0.001 | <0.001 |
| **GM-CSF** | 0.027 | 0.096 | 0.134 | -0.099 | -0.04 | -0.072 | -0.174 | 0.002 |
| *p-value* | 0.698 | 0.162 | 0.052 | 0.147 | 0.555 | 0.313 | 0.014 | 0.978 |
| **GRO-alpha** | 0.206 | -0.026 | -0.03 | 0.002 | -0.148 | -0.027 | -0.117 | -0.192 |
| *p-value* | 0.009 | 0.744 | 0.712 | 0.975 | 0.063 | 0.741 | 0.15 | 0.018 |
| **HGF** | -0.387 | 0.504 | 0.453 | 0.173 | -0.148 | 0.063 | -0.216 | -0.237 |
| *p-value* | <0.001 | <0.001 | <0.001 | 0.003 | 0.01 | 0.296 | <0.001 | <0.001 |
| **IFN-alpha** | 0.156 | 0.018 | 0.086 | -0.048 | -0.218 | -0.218 | -0.221 | -0.106 |
| *p-value* | 0.009 | 0.767 | 0.153 | 0.418 | <0.001 | <0.001 | <0.001 | 0.089 |
| **IFN-gamma** | 0.167 | -0.008 | 0.041 | -0.117 | -0.162 | -0.3 | -0.18 | 0.025 |
| *p-value* | 0.004 | 0.896 | 0.482 | 0.044 | 0.005 | <0.001 | 0.003 | 0.687 |
| **IL-1-alpha** | -0.316 | 0.414 | 0.362 | 0.1 | -0.278 | 0.028 | -0.306 | -0.364 |
| *p-value* | <0.001 | <0.001 | <0.001 | 0.085 | <0.001 | 0.648 | <0.001 | <0.001 |
| **IL-1-beta** | -0.162 | 0.304 | 0.303 | 0.035 | -0.141 | -0.039 | -0.195 | -0.167 |
| *p-value* | 0.005 | <0.001 | <0.001 | 0.543 | 0.015 | 0.52 | 0.001 | 0.006 |
| **IL-1RA** | 0.26 | -0.114 | -0.033 | -0.175 | -0.191 | -0.481 | -0.116 | 0.155 |
| *p-value* | <0.001 | 0.051 | 0.574 | 0.002 | 0.001 | <0.001 | 0.055 | 0.011 |
| **IL-2** | 0.031 | 0.139 | 0.189 | -0.05 | -0.236 | -0.059 | -0.257 | -0.242 |
| *p-value* | 0.648 | 0.039 | 0.005 | 0.453 | <0.001 | 0.406 | <0.001 | 0.001 |
| **IL2-R-alpha** | -0.19 | 0.297 | 0.288 | 0.052 | -0.157 | 0.043 | -0.23 | -0.231 |
| *p-value* | 0.001 | <0.001 | <0.001 | 0.37 | 0.007 | 0.483 | <0.001 | <0.001 |
| **IL-3** | -0.192 | 0.341 | 0.277 | -0.062 | -0.159 | 0.008 | -0.217 | -0.263 |
| *p-value* | 0.016 | <0.001 | 0.001 | 0.439 | 0.048 | 0.924 | 0.01 | 0.002 |
| **IL-4** | 0.024 | 0.168 | 0.218 | -0.029 | -0.195 | -0.079 | -0.219 | -0.17 |
| *p-value* | 0.684 | 0.004 | <0.001 | 0.619 | 0.001 | 0.192 | <0.001 | 0.005 |
| **IL-5** | 0.268 | -0.188 | -0.138 | -0.069 | 0.061 | -0.152 | -0.055 | 0.245 |
| *p-value* | 0.037 | 0.15 | 0.292 | 0.595 | 0.639 | 0.263 | 0.686 | 0.068 |
| **IL-6** | 0.092 | 0.017 | 0.095 | -0.136 | 0.013 | -0.15 | -0.05 | 0.155 |
| *p-value* | 0.232 | 0.823 | 0.223 | 0.077 | 0.866 | 0.062 | 0.539 | 0.055 |
| **IL-7** | 0.115 | -0.056 | -0.044 | -0.162 | -0.047 | -0.107 | -0.035 | 0.014 |
| *p-value* | 0.059 | 0.36 | 0.479 | 0.007 | 0.445 | 0.092 | 0.585 | 0.827 |
| **IL-8** | -0.255 | 0.367 | 0.334 | 0.084 | -0.238 | -0.09 | -0.295 | -0.197 |
| *p-value* | <0.001 | <0.001 | <0.001 | 0.148 | <0.001 | 0.135 | <0.001 | 0.001 |
| **IL-9** | 0.457 | -0.275 | -0.158 | -0.137 | -0.1 | -0.105 | -0.125 | 0.001 |
| *p-value* | <0.001 | <0.001 | 0.007 | 0.018 | 0.083 | 0.084 | 0.038 | 0.984 |
| **IL-10** | -0.26 | 0.357 | 0.311 | 0.053 | -0.292 | -0.181 | -0.339 | -0.292 |
| *p-value* | <0.001 | <0.001 | <0.001 | 0.368 | <0.001 | 0.003 | <0.001 | <0.001 |
| **IL-12p70** | -0.062 | 0.162 | 0.11 | -0.092 | -0.136 | -0.095 | -0.161 | -0.086 |
| *p-value* | 0.329 | 0.011 | 0.087 | 0.147 | 0.032 | 0.148 | 0.014 | 0.193 |
| **IL-12p40** | -0.195 | 0.363 | 0.367 | 0.007 | -0.147 | 0.043 | -0.154 | -0.226 |
| *p-value* | 0.001 | <0.001 | <0.001 | 0.908 | 0.012 | 0.491 | 0.012 | <0.001 |
| **IL-13** | -0.218 | 0.299 | 0.261 | 0.064 | -0.101 | 0.09 | -0.118 | -0.203 |
| *p-value* | <0.001 | <0.001 | <0.001 | 0.273 | 0.081 | 0.137 | 0.051 | 0.001 |
| **IL-15** | 0.273 | -0.125 | -0.19 | -0.284 | -0.034 | -0.085 | -0.113 | 0.041 |
| *p-value* | 0.05 | 0.381 | 0.182 | 0.041 | 0.812 | 0.576 | 0.455 | 0.788 |
| **IL-16** | -0.198 | 0.284 | 0.257 | 0.087 | -0.239 | -0.08 | -0.327 | -0.225 |
| *p-value* | 0.001 | <0.001 | <0.001 | 0.133 | <0.001 | 0.187 | <0.001 | <0.001 |
| **IL-17** | -0.259 | 0.394 | 0.36 | 0.046 | -0.271 | -0.012 | -0.32 | -0.331 |
| *p-value* | <0.001 | <0.001 | <0.001 | 0.433 | <0.001 | 0.846 | <0.001 | <0.001 |
| **IL-18** | -0.199 | 0.308 | 0.315 | 0.155 | -0.226 | -0.03 | -0.256 | -0.211 |
| *p-value* | 0.001 | <0.001 | <0.001 | 0.007 | <0.001 | 0.621 | <0.001 | <0.001 |
| **IP10** | -0.156 | 0.279 | 0.267 | 0.066 | -0.323 | -0.192 | -0.35 | -0.285 |
| *p-value* | 0.007 | <0.001 | <0.001 | 0.259 | <0.001 | 0.001 | <0.001 | <0.001 |
| **LIF** | -0.299 | 0.404 | 0.367 | 0.117 | -0.288 | -0.005 | -0.301 | -0.341 |
| *p-value* | <0.001 | <0.001 | <0.001 | 0.043 | <0.001 | 0.932 | <0.001 | <0.001 |
| **MCP1/MCAF** | 0.148 | 0.004 | 0.072 | -0.085 | -0.284 | -0.348 | -0.276 | -0.076 |
| *p-value* | 0.01 | 0.95 | 0.22 | 0.144 | <0.001 | <0.001 | <0.001 | 0.207 |
| **MCP3** | -0.076 | 0.212 | 0.224 | -0.015 | -0.277 | -0.194 | -0.315 | -0.199 |
| *p-value* | 0.198 | <0.001 | <0.001 | 0.795 | <0.001 | 0.002 | <0.001 | 0.001 |
| **M-CSF** | -0.111 | 0.228 | 0.232 | 0.047 | -0.243 | -0.189 | -0.284 | -0.163 |
| *p-value* | 0.055 | <0.001 | <0.001 | 0.42 | <0.001 | 0.002 | <0.001 | 0.007 |
| **MIF** | -0.058 | 0.214 | 0.219 | 0.051 | -0.082 | -0.053 | -0.141 | -0.074 |
| *p-value* | 0.32 | <0.001 | <0.001 | 0.383 | 0.162 | 0.389 | 0.02 | 0.226 |
| **MIG** | -0.304 | 0.378 | 0.345 | 0.123 | -0.222 | 0.066 | -0.294 | -0.324 |
| *p-value* | <0.001 | <0.001 | <0.001 | 0.034 | <0.001 | 0.279 | <0.001 | <0.001 |
| **MIP1-alpha** | -0.242 | 0.336 | 0.298 | 0.06 | -0.356 | -0.145 | -0.427 | -0.34 |
| *p-value* | <0.001 | <0.001 | <0.001 | 0.3 | <0.001 | 0.016 | <0.001 | <0.001 |
| **MIP1-beta** | 0.378 | -0.203 | -0.092 | -0.122 | -0.142 | -0.136 | -0.176 | -0.025 |
| *p-value* | <0.001 | <0.001 | 0.117 | 0.035 | 0.014 | 0.024 | 0.003 | 0.682 |
| **Beta-NGF** | -0.034 | 0.157 | 0.168 | 0.016 | -0.236 | 0.03 | -0.271 | -0.27 |
| *p-value* | 0.672 | 0.051 | 0.036 | 0.84 | 0.003 | 0.719 | 0.001 | 0.001 |
| **PDGF-BB** | -0.007 | 0.205 | 0.203 | 0.099 | -0.183 | -0.01 | -0.186 | -0.212 |
| *p-value* | 0.909 | 0.001 | 0.001 | 0.122 | 0.004 | 0.882 | 0.005 | 0.001 |
| **RANTES** | 0.38 | -0.23 | -0.145 | -0.097 | -0.113 | -0.08 | -0.133 | -0.031 |
| *p-value* | <0.001 | <0.001 | 0.015 | 0.102 | 0.055 | 0.199 | 0.032 | 0.62 |
| **SCF** | -0.165 | 0.273 | 0.268 | 0.054 | -0.213 | -0.03 | -0.241 | -0.233 |
| *p-value* | 0.004 | <0.001 | <0.001 | 0.348 | <0.001 | 0.618 | <0.001 | <0.001 |
| **SCGF-beta** | -0.485 | 0.52 | 0.443 | 0.244 | -0.219 | 0.134 | -0.261 | -0.369 |
| *p-value* | <0.001 | <0.001 | <0.001 | <0.001 | <0.001 | 0.027 | <0.001 | <0.001 |
| **SDF-1-alpha** | -0.059 | 0.154 | 0.157 | 0.111 | -0.228 | 0.022 | -0.258 | -0.291 |
| *p-value* | 0.313 | 0.008 | 0.007 | 0.054 | <0.001 | 0.712 | <0.001 | <0.001 |
| **TNF-alpha** | -0.083 | 0.227 | 0.229 | 0.044 | -0.246 | -0.049 | -0.278 | -0.279 |
| *p-value* | 0.153 | <0.001 | <0.001 | 0.448 | <0.001 | 0.422 | <0.001 | <0.001 |
| **TNF-beta** | 0.478 | -0.295 | -0.17 | -0.157 | -0.086 | -0.119 | -0.111 | 0.039 |
| *p-value* | <0.001 | <0.001 | 0.004 | 0.006 | 0.14 | 0.049 | 0.066 | 0.52 |
| **TRAIL** | -0.141 | 0.231 | 0.215 | 0.08 | -0.317 | -0.095 | -0.292 | -0.317 |
| *p-value* | 0.015 | <0.001 | <0.001 | 0.166 | <0.001 | 0.115 | <0.001 | <0.001 |
| **VEGF** | 0.17 | -0.068 | -0.04 | 0.047 | -0.003 | 0.026 | 0.021 | -0.08 |
| *p-value* | 0.093 | 0.504 | 0.694 | 0.642 | 0.979 | 0.803 | 0.84 | 0.447 |

The names of inflammatory mediators are detailed in the abbreviation list. PLT: platelet count, AST: aspartate aminotransferase, ALT: alanine aminotransferase, HCT: haematocrit, WBC: leucocytes count, LYM: lymphocytes count, MONO: monocytes count, NEU: neutrophils count. The correlation coefficient was calculated using Spearman's method with Holm corrections. P-value <0.05 is considered significant.

# **Supplementary Table S3. Correlation matrix of plasma levels of inflammatory mediators in dengue patients**

|  | **CTACK** | **Eotaxin** | **FGF-basic** | **G-CSF** | **GM-CSF** | **GRO-alpha** | **HGF** | **IFN-alpha** | **IFN-gamma** | **IL-1-alpha** | **IL-1-beta** | **IL-1RA** | **IL-2** | **IL-2R-alpha** | **IL-3** | **IL-4** |
| --- | --- | --- | --- | --- | --- | --- | --- | --- | --- | --- | --- | --- | --- | --- | --- | --- |
| **CTACK** | 1 | 0.806 | 0.904 | 0.765 | 0.324 | 0.539 | 0.881 | 0.522 | 0.534 | 0.815 | 0.591 | 0.349 | 0.533 | 0.83 | 0.715 | 0.544 |
| *p-value* | <0.001 | <0.001 | <0.001 | <0.001 | <0.001 | <0.001 | <0.001 | <0.001 | <0.001 | <0.001 | <0.001 | <0.001 | <0.001 | <0.001 | <0.001 | <0.001 |
| **Eotaxin** | 0.806 | 1 | 0.769 | 0.724 | 0.342 | 0.443 | 0.801 | 0.548 | 0.594 | 0.707 | 0.606 | 0.452 | 0.517 | 0.726 | 0.636 | 0.575 |
| *p-value* | <0.001 | <0.001 | <0.001 | <0.001 | <0.001 | <0.001 | <0.001 | <0.001 | <0.001 | <0.001 | <0.001 | <0.001 | <0.001 | <0.001 | <0.001 | <0.001 |
| **FGF-basic** | 0.904 | 0.769 | 1 | 0.782 | 0.315 | 0.655 | 0.85 | 0.53 | 0.602 | 0.876 | 0.522 | 0.384 | 0.595 | 0.804 | 0.775 | 0.523 |
| *p-value* | <0.001 | <0.001 | <0.001 | <0.001 | <0.001 | <0.001 | <0.001 | <0.001 | <0.001 | <0.001 | <0.001 | <0.001 | <0.001 | <0.001 | <0.001 | <0.001 |
| **G-CSF** | 0.765 | 0.724 | 0.782 | 1 | 0.44 | 0.546 | 0.769 | 0.631 | 0.718 | 0.764 | 0.639 | 0.586 | 0.596 | 0.786 | 0.691 | 0.679 |
| *p-value* | <0.001 | <0.001 | <0.001 | <0.001 | <0.001 | <0.001 | <0.001 | <0.001 | <0.001 | <0.001 | <0.001 | <0.001 | <0.001 | <0.001 | <0.001 | <0.001 |
| **GM-CSF** | 0.324 | 0.342 | 0.315 | 0.44 | 1 | 0.193 | 0.352 | 0.488 | 0.384 | 0.353 | 0.588 | 0.297 | 0.493 | 0.434 | 0.317 | 0.603 |
| *p-value* | <0.001 | <0.001 | <0.001 | <0.001 | <0.001 | 0.028 | <0.001 | <0.001 | <0.001 | <0.001 | <0.001 | <0.001 | <0.001 | <0.001 | <0.001 | <0.001 |
| **GRO-alpha** | 0.539 | 0.443 | 0.655 | 0.546 | 0.193 | 1 | 0.363 | 0.402 | 0.519 | 0.439 | 0.147 | 0.279 | 0.534 | 0.537 | 0.387 | 0.425 |
| *p-value* | <0.001 | <0.001 | <0.001 | <0.001 | 0.028 | <0.001 | <0.001 | <0.001 | <0.001 | <0.001 | 0.065 | <0.001 | <0.001 | <0.001 | <0.001 | <0.001 |
| **HGF** | 0.881 | 0.801 | 0.85 | 0.769 | 0.352 | 0.363 | 1 | 0.476 | 0.544 | 0.817 | 0.661 | 0.4 | 0.521 | 0.866 | 0.725 | 0.548 |
| *p-value* | <0.001 | <0.001 | <0.001 | <0.001 | <0.001 | <0.001 | <0.001 | <0.001 | <0.001 | <0.001 | <0.001 | <0.001 | <0.001 | <0.001 | <0.001 | <0.001 |
| **IFN-alpha** | 0.522 | 0.548 | 0.53 | 0.631 | 0.488 | 0.402 | 0.476 | 1 | 0.669 | 0.539 | 0.556 | 0.619 | 0.719 | 0.561 | 0.471 | 0.777 |
| *p-value* | <0.001 | <0.001 | <0.001 | <0.001 | <0.001 | <0.001 | <0.001 | <0.001 | <0.001 | <0.001 | <0.001 | <0.001 | <0.001 | <0.001 | <0.001 | <0.001 |
| **IFN-gamma** | 0.534 | 0.594 | 0.602 | 0.718 | 0.384 | 0.519 | 0.544 | 0.669 | 1 | 0.511 | 0.466 | 0.8 | 0.516 | 0.666 | 0.479 | 0.56 |
| *p-value* | <0.001 | <0.001 | <0.001 | <0.001 | <0.001 | <0.001 | <0.001 | <0.001 | <0.001 | <0.001 | <0.001 | <0.001 | <0.001 | <0.001 | <0.001 | <0.001 |
| **IL-1-alpha** | 0.815 | 0.707 | 0.876 | 0.764 | 0.353 | 0.439 | 0.817 | 0.539 | 0.511 | 1 | 0.62 | 0.326 | 0.548 | 0.748 | 0.79 | 0.587 |
| *p-value* | <0.001 | <0.001 | <0.001 | <0.001 | <0.001 | <0.001 | <0.001 | <0.001 | <0.001 | <0.001 | <0.001 | <0.001 | <0.001 | <0.001 | <0.001 | <0.001 |
| **IL-1-beta** | 0.591 | 0.606 | 0.522 | 0.639 | 0.588 | 0.147 | 0.661 | 0.556 | 0.466 | 0.62 | 1 | 0.343 | 0.528 | 0.635 | 0.468 | 0.665 |
| *p-value* | <0.001 | <0.001 | <0.001 | <0.001 | <0.001 | 0.065 | <0.001 | <0.001 | <0.001 | <0.001 | <0.001 | <0.001 | <0.001 | <0.001 | <0.001 | <0.001 |
| **IL-1RA** | 0.349 | 0.452 | 0.384 | 0.586 | 0.297 | 0.279 | 0.4 | 0.619 | 0.8 | 0.326 | 0.343 | 1 | 0.457 | 0.467 | 0.347 | 0.455 |
| *p-value* | <0.001 | <0.001 | <0.001 | <0.001 | <0.001 | <0.001 | <0.001 | <0.001 | <0.001 | <0.001 | <0.001 | <0.001 | <0.001 | <0.001 | <0.001 | <0.001 |
| **IL-2** | 0.533 | 0.517 | 0.595 | 0.596 | 0.493 | 0.534 | 0.521 | 0.719 | 0.516 | 0.548 | 0.528 | 0.457 | 1 | 0.59 | 0.532 | 0.803 |
| *p-value* | <0.001 | <0.001 | <0.001 | <0.001 | <0.001 | <0.001 | <0.001 | <0.001 | <0.001 | <0.001 | <0.001 | <0.001 | <0.001 | <0.001 | <0.001 | <0.001 |
| **IL2-R-alpha** | 0.83 | 0.726 | 0.804 | 0.786 | 0.434 | 0.537 | 0.866 | 0.561 | 0.666 | 0.748 | 0.635 | 0.467 | 0.59 | 1 | 0.659 | 0.618 |
| *p-value* | <0.001 | <0.001 | <0.001 | <0.001 | <0.001 | <0.001 | <0.001 | <0.001 | <0.001 | <0.001 | <0.001 | <0.001 | <0.001 | <0.001 | <0.001 | <0.001 |
| **IL-3** | 0.715 | 0.636 | 0.775 | 0.691 | 0.317 | 0.387 | 0.725 | 0.471 | 0.479 | 0.79 | 0.468 | 0.347 | 0.532 | 0.659 | 1 | 0.427 |
| *p-value* | <0.001 | <0.001 | <0.001 | <0.001 | <0.001 | <0.001 | <0.001 | <0.001 | <0.001 | <0.001 | <0.001 | <0.001 | <0.001 | <0.001 | <0.001 | <0.001 |
| **IL-4** | 0.544 | 0.575 | 0.523 | 0.679 | 0.603 | 0.425 | 0.548 | 0.777 | 0.56 | 0.587 | 0.665 | 0.455 | 0.803 | 0.618 | 0.427 | 1 |
| *p-value* | <0.001 | <0.001 | <0.001 | <0.001 | <0.001 | <0.001 | <0.001 | <0.001 | <0.001 | <0.001 | <0.001 | <0.001 | <0.001 | <0.001 | <0.001 | <0.001 |
| **IL-5** | -0.103 | 0.156 | -0.03 | 0.039 | 0.538 | 0.167 | -0.066 | 0.361 | 0.227 | -0.1 | 0.103 | 0.22 | 0.466 | 0.09 | -0.144 | 0.541 |
| *p-value* | 0.433 | 0.229 | 0.821 | 0.763 | <0.001 | 0.231 | 0.615 | 0.005 | 0.079 | 0.445 | 0.429 | 0.089 | <0.001 | 0.489 | 0.318 | <0.001 |
| **IL-6** | 0.208 | 0.293 | 0.3 | 0.364 | 0.413 | 0.273 | 0.337 | 0.385 | 0.496 | 0.295 | 0.249 | 0.499 | 0.436 | 0.374 | 0.127 | 0.451 |
| *p-value* | 0.007 | <0.001 | <0.001 | <0.001 | <0.001 | 0.003 | <0.001 | <0.001 | <0.001 | <0.001 | 0.001 | <0.001 | <0.001 | <0.001 | 0.181 | <0.001 |
| **IL-7** | -0.094 | 0.068 | -0.091 | 0.137 | 0.297 | 0.011 | -0.021 | 0.321 | 0.045 | 0.095 | 0.283 | 0.083 | 0.306 | 0.012 | -0.108 | 0.553 |
| *p-value* | 0.125 | 0.261 | 0.138 | 0.024 | <0.001 | 0.891 | 0.732 | <0.001 | 0.466 | 0.12 | <0.001 | 0.174 | <0.001 | 0.847 | 0.193 | <0.001 |
| **IL-8** | 0.737 | 0.74 | 0.724 | 0.813 | 0.478 | 0.354 | 0.825 | 0.597 | 0.615 | 0.726 | 0.675 | 0.556 | 0.623 | 0.768 | 0.621 | 0.696 |
| *p-value* | <0.001 | <0.001 | <0.001 | <0.001 | <0.001 | <0.001 | <0.001 | <0.001 | <0.001 | <0.001 | <0.001 | <0.001 | <0.001 | <0.001 | <0.001 | <0.001 |
| **IL-9** | 0.376 | 0.347 | 0.412 | 0.491 | 0.422 | 0.745 | 0.3 | 0.617 | 0.602 | 0.3 | 0.346 | 0.475 | 0.572 | 0.481 | 0.336 | 0.559 |
| *p-value* | <0.001 | <0.001 | <0.001 | <0.001 | <0.001 | <0.001 | <0.001 | <0.001 | <0.001 | <0.001 | <0.001 | <0.001 | <0.001 | <0.001 | <0.001 | <0.001 |
| **IL-10** | 0.638 | 0.561 | 0.611 | 0.707 | 0.357 | 0.303 | 0.685 | 0.458 | 0.481 | 0.619 | 0.548 | 0.409 | 0.567 | 0.646 | 0.568 | 0.586 |
| *p-value* | <0.001 | <0.001 | <0.001 | <0.001 | <0.001 | <0.001 | <0.001 | <0.001 | <0.001 | <0.001 | <0.001 | <0.001 | <0.001 | <0.001 | <0.001 | <0.001 |
| **IL-12p70** | 0.389 | 0.434 | 0.375 | 0.476 | 0.573 | 0.131 | 0.453 | 0.45 | 0.35 | 0.49 | 0.563 | 0.322 | 0.54 | 0.483 | 0.378 | 0.583 |
| *p-value* | <0.001 | <0.001 | <0.001 | <0.001 | <0.001 | 0.113 | <0.001 | <0.001 | <0.001 | <0.001 | <0.001 | <0.001 | <0.001 | <0.001 | <0.001 | <0.001 |
| **IL-12p40** | 0.652 | 0.624 | 0.705 | 0.676 | 0.387 | 0.416 | 0.738 | 0.558 | 0.477 | 0.796 | 0.592 | 0.343 | 0.649 | 0.685 | 0.781 | 0.673 |
| *p-value* | <0.001 | <0.001 | <0.001 | <0.001 | <0.001 | <0.001 | <0.001 | <0.001 | <0.001 | <0.001 | <0.001 | <0.001 | <0.001 | <0.001 | <0.001 | <0.001 |
| **IL-13** | 0.417 | 0.472 | 0.384 | 0.442 | 0.465 | -0.017 | 0.517 | 0.337 | 0.246 | 0.541 | 0.771 | 0.113 | 0.358 | 0.468 | 0.401 | 0.489 |
| *p-value* | <0.001 | <0.001 | <0.001 | <0.001 | <0.001 | 0.832 | <0.001 | <0.001 | <0.001 | <0.001 | <0.001 | 0.053 | <0.001 | <0.001 | <0.001 | <0.001 |
| **IL-15** | 0.322 | 0.302 | 0.466 | 0.369 | 0.478 | 0.624 | 0.401 | 0.498 | 0.421 | 0.383 | 0.426 | 0.319 | 0.67 | 0.44 | 0.198 | 0.569 |
| *p-value* | 0.02 | 0.03 | <0.001 | 0.007 | 0.001 | <0.001 | 0.003 | <0.001 | 0.002 | 0.006 | 0.002 | 0.021 | <0.001 | 0.001 | 0.21 | <0.001 |
| **IL-16** | 0.834 | 0.717 | 0.799 | 0.822 | 0.53 | 0.48 | 0.842 | 0.626 | 0.648 | 0.735 | 0.661 | 0.547 | 0.605 | 0.877 | 0.627 | 0.658 |
| *p-value* | <0.001 | <0.001 | <0.001 | <0.001 | <0.001 | <0.001 | <0.001 | <0.001 | <0.001 | <0.001 | <0.001 | <0.001 | <0.001 | <0.001 | <0.001 | <0.001 |
| **IL-17** | 0.774 | 0.736 | 0.816 | 0.81 | 0.464 | 0.415 | 0.798 | 0.599 | 0.561 | 0.875 | 0.668 | 0.37 | 0.61 | 0.753 | 0.735 | 0.724 |
| *p-value* | <0.001 | <0.001 | <0.001 | <0.001 | <0.001 | <0.001 | <0.001 | <0.001 | <0.001 | <0.001 | <0.001 | <0.001 | <0.001 | <0.001 | <0.001 | <0.001 |
| **IL-18** | 0.801 | 0.687 | 0.76 | 0.771 | 0.376 | 0.459 | 0.821 | 0.573 | 0.595 | 0.686 | 0.603 | 0.504 | 0.585 | 0.83 | 0.587 | 0.627 |
| *p-value* | <0.001 | <0.001 | <0.001 | <0.001 | <0.001 | <0.001 | <0.001 | <0.001 | <0.001 | <0.001 | <0.001 | <0.001 | <0.001 | <0.001 | <0.001 | <0.001 |
| **IP10** | 0.83 | 0.8 | 0.797 | 0.785 | 0.42 | 0.539 | 0.764 | 0.664 | 0.714 | 0.676 | 0.563 | 0.631 | 0.6 | 0.776 | 0.629 | 0.627 |
| *p-value* | <0.001 | <0.001 | <0.001 | <0.001 | <0.001 | <0.001 | <0.001 | <0.001 | <0.001 | <0.001 | <0.001 | <0.001 | <0.001 | <0.001 | <0.001 | <0.001 |
| **LIF** | 0.874 | 0.776 | 0.919 | 0.806 | 0.412 | 0.515 | 0.869 | 0.582 | 0.594 | 0.916 | 0.612 | 0.416 | 0.644 | 0.829 | 0.817 | 0.636 |
| *p-value* | <0.001 | <0.001 | <0.001 | <0.001 | <0.001 | <0.001 | <0.001 | <0.001 | <0.001 | <0.001 | <0.001 | <0.001 | <0.001 | <0.001 | <0.001 | <0.001 |
| **MCP1/MCAF** | 0.525 | 0.623 | 0.521 | 0.673 | 0.424 | 0.348 | 0.482 | 0.678 | 0.79 | 0.442 | 0.509 | 0.764 | 0.577 | 0.537 | 0.402 | 0.614 |
| *p-value* | <0.001 | <0.001 | <0.001 | <0.001 | <0.001 | <0.001 | <0.001 | <0.001 | <0.001 | <0.001 | <0.001 | <0.001 | <0.001 | <0.001 | <0.001 | <0.001 |
| **MCP3** | 0.745 | 0.779 | 0.785 | 0.863 | 0.417 | 0.637 | 0.734 | 0.742 | 0.804 | 0.736 | 0.609 | 0.695 | 0.665 | 0.75 | 0.673 | 0.717 |
| *p-value* | <0.001 | <0.001 | <0.001 | <0.001 | <0.001 | <0.001 | <0.001 | <0.001 | <0.001 | <0.001 | <0.001 | <0.001 | <0.001 | <0.001 | <0.001 | <0.001 |
| **M-CSF** | 0.774 | 0.705 | 0.766 | 0.786 | 0.42 | 0.497 | 0.811 | 0.637 | 0.737 | 0.698 | 0.617 | 0.66 | 0.591 | 0.868 | 0.606 | 0.594 |
| *p-value* | <0.001 | <0.001 | <0.001 | <0.001 | <0.001 | <0.001 | <0.001 | <0.001 | <0.001 | <0.001 | <0.001 | <0.001 | <0.001 | <0.001 | <0.001 | <0.001 |
| **MIF** | 0.637 | 0.67 | 0.56 | 0.67 | 0.427 | 0.318 | 0.688 | 0.599 | 0.578 | 0.548 | 0.639 | 0.522 | 0.485 | 0.696 | 0.56 | 0.608 |
| *p-value* | <0.001 | <0.001 | <0.001 | <0.001 | <0.001 | <0.001 | <0.001 | <0.001 | <0.001 | <0.001 | <0.001 | <0.001 | <0.001 | <0.001 | <0.001 | <0.001 |
| **MIG** | 0.882 | 0.78 | 0.871 | 0.779 | 0.381 | 0.536 | 0.846 | 0.478 | 0.611 | 0.774 | 0.528 | 0.399 | 0.568 | 0.834 | 0.723 | 0.541 |
| *p-value* | <0.001 | <0.001 | <0.001 | <0.001 | <0.001 | <0.001 | <0.001 | <0.001 | <0.001 | <0.001 | <0.001 | <0.001 | <0.001 | <0.001 | <0.001 | <0.001 |
| **MIP1-alpha** | 0.802 | 0.724 | 0.804 | 0.913 | 0.43 | 0.394 | 0.827 | 0.541 | 0.624 | 0.79 | 0.643 | 0.542 | 0.571 | 0.803 | 0.661 | 0.637 |
| *p-value* | <0.001 | <0.001 | <0.001 | <0.001 | <0.001 | <0.001 | <0.001 | <0.001 | <0.001 | <0.001 | <0.001 | <0.001 | <0.001 | <0.001 | <0.001 | <0.001 |
| **MIP1-beta** | 0.491 | 0.478 | 0.523 | 0.629 | 0.488 | 0.68 | 0.448 | 0.688 | 0.694 | 0.403 | 0.459 | 0.604 | 0.618 | 0.604 | 0.36 | 0.652 |
| *p-value* | <0.001 | <0.001 | <0.001 | <0.001 | <0.001 | <0.001 | <0.001 | <0.001 | <0.001 | <0.001 | <0.001 | <0.001 | <0.001 | <0.001 | <0.001 | <0.001 |
| **Beta-NGF** | 0.776 | 0.665 | 0.837 | 0.723 | 0.423 | 0.789 | 0.747 | 0.541 | 0.566 | 0.699 | 0.474 | 0.393 | 0.715 | 0.797 | 0.609 | 0.532 |
| *p-value* | <0.001 | <0.001 | <0.001 | <0.001 | <0.001 | <0.001 | <0.001 | <0.001 | <0.001 | <0.001 | <0.001 | <0.001 | <0.001 | <0.001 | <0.001 | <0.001 |
| **PDGF-BB** | 0.673 | 0.538 | 0.729 | 0.565 | 0.215 | 0.689 | 0.546 | 0.468 | 0.467 | 0.569 | 0.173 | 0.278 | 0.426 | 0.529 | 0.464 | 0.44 |
| *p-value* | <0.001 | <0.001 | <0.001 | <0.001 | 0.003 | <0.001 | <0.001 | <0.001 | <0.001 | <0.001 | 0.007 | <0.001 | <0.001 | <0.001 | <0.001 | <0.001 |
| **RANTES** | 0.452 | 0.385 | 0.48 | 0.495 | 0.355 | 0.663 | 0.369 | 0.556 | 0.577 | 0.367 | 0.322 | 0.477 | 0.463 | 0.514 | 0.405 | 0.436 |
| *p-value* | <0.001 | <0.001 | <0.001 | <0.001 | <0.001 | <0.001 | <0.001 | <0.001 | <0.001 | <0.001 | <0.001 | <0.001 | <0.001 | <0.001 | <0.001 | <0.001 |
| **SCF** | 0.843 | 0.756 | 0.839 | 0.797 | 0.428 | 0.586 | 0.847 | 0.622 | 0.664 | 0.786 | 0.662 | 0.521 | 0.628 | 0.884 | 0.679 | 0.659 |
| *p-value* | <0.001 | <0.001 | <0.001 | <0.001 | <0.001 | <0.001 | <0.001 | <0.001 | <0.001 | <0.001 | <0.001 | <0.001 | <0.001 | <0.001 | <0.001 | <0.001 |
| **SCGF-beta** | 0.882 | 0.778 | 0.825 | 0.675 | 0.318 | 0.272 | 0.898 | 0.41 | 0.417 | 0.787 | 0.609 | 0.279 | 0.489 | 0.8 | 0.648 | 0.5 |
| *p-value* | <0.001 | <0.001 | <0.001 | <0.001 | <0.001 | 0.001 | <0.001 | <0.001 | <0.001 | <0.001 | <0.001 | <0.001 | <0.001 | <0.001 | <0.001 | <0.001 |
| **SDF-1-alpha** | 0.821 | 0.743 | 0.828 | 0.742 | 0.371 | 0.822 | 0.723 | 0.636 | 0.65 | 0.627 | 0.493 | 0.472 | 0.608 | 0.761 | 0.577 | 0.641 |
| *p-value* | <0.001 | <0.001 | <0.001 | <0.001 | <0.001 | <0.001 | <0.001 | <0.001 | <0.001 | <0.001 | <0.001 | <0.001 | <0.001 | <0.001 | <0.001 | <0.001 |
| **TNF-alpha** | 0.775 | 0.708 | 0.832 | 0.816 | 0.474 | 0.617 | 0.757 | 0.628 | 0.632 | 0.827 | 0.652 | 0.466 | 0.607 | 0.768 | 0.734 | 0.665 |
| *p-value* | <0.001 | <0.001 | <0.001 | <0.001 | <0.001 | <0.001 | <0.001 | <0.001 | <0.001 | <0.001 | <0.001 | <0.001 | <0.001 | <0.001 | <0.001 | <0.001 |
| **TNF-beta** | 0.343 | 0.325 | 0.368 | 0.466 | 0.452 | 0.657 | 0.285 | 0.597 | 0.576 | 0.276 | 0.359 | 0.488 | 0.548 | 0.468 | 0.3 | 0.552 |
| *p-value* | <0.001 | <0.001 | <0.001 | <0.001 | <0.001 | <0.001 | <0.001 | <0.001 | <0.001 | <0.001 | <0.001 | <0.001 | <0.001 | <0.001 | <0.001 | <0.001 |
| **TRAIL** | 0.839 | 0.764 | 0.909 | 0.777 | 0.292 | 0.619 | 0.811 | 0.57 | 0.706 | 0.8 | 0.535 | 0.558 | 0.471 | 0.803 | 0.713 | 0.483 |
| *p-value* | <0.001 | <0.001 | <0.001 | <0.001 | <0.001 | <0.001 | <0.001 | <0.001 | <0.001 | <0.001 | <0.001 | <0.001 | <0.001 | <0.001 | <0.001 | <0.001 |
| **VEGF** | 0.236 | 0.157 | 0.307 | 0.343 | 0.318 | 0.577 | 0.152 | 0.35 | 0.34 | 0.191 | 0.159 | 0.24 | 0.632 | 0.301 | 0.205 | 0.512 |
| *p-value* | 0.019 | 0.121 | 0.002 | 0.001 | 0.002 | <0.001 | 0.133 | 0.001 | 0.001 | 0.059 | 0.116 | 0.017 | <0.001 | 0.002 | 0.074 | <0.001 |

|  | **IL-5** | **IL-6** | **IL-7** | **IL-8** | **IL-9** | **IL-10** | **IL-12p70** | **IL-12p40** | **IL-13** | **IL-15** | **IL-16** | **IL-17** | **IL-18** | **IP10** | **LIF** | **MCP1/MCAF** |
| --- | --- | --- | --- | --- | --- | --- | --- | --- | --- | --- | --- | --- | --- | --- | --- | --- |
| **CTACK** | -0.103 | 0.208 | -0.094 | 0.737 | 0.376 | 0.638 | 0.389 | 0.652 | 0.417 | 0.322 | 0.834 | 0.774 | 0.801 | 0.83 | 0.874 | 0.525 |
| *p-value* | 0.433 | 0.007 | 0.125 | <0.001 | <0.001 | <0.001 | <0.001 | <0.001 | <0.001 | 0.02 | <0.001 | <0.001 | <0.001 | <0.001 | <0.001 | <0.001 |
| **Eotaxin** | 0.156 | 0.293 | 0.068 | 0.74 | 0.347 | 0.561 | 0.434 | 0.624 | 0.472 | 0.302 | 0.717 | 0.736 | 0.687 | 0.8 | 0.776 | 0.623 |
| *p-value* | 0.229 | <0.001 | 0.261 | <0.001 | <0.001 | <0.001 | <0.001 | <0.001 | <0.001 | 0.03 | <0.001 | <0.001 | <0.001 | <0.001 | <0.001 | <0.001 |
| **FGF-basic** | -0.03 | 0.3 | -0.091 | 0.724 | 0.412 | 0.611 | 0.375 | 0.705 | 0.384 | 0.466 | 0.799 | 0.816 | 0.76 | 0.797 | 0.919 | 0.521 |
| *p-value* | 0.821 | <0.001 | 0.138 | <0.001 | <0.001 | <0.001 | <0.001 | <0.001 | <0.001 | <0.001 | <0.001 | <0.001 | <0.001 | <0.001 | <0.001 | <0.001 |
| **G-CSF** | 0.039 | 0.364 | 0.137 | 0.813 | 0.491 | 0.707 | 0.476 | 0.676 | 0.442 | 0.369 | 0.822 | 0.81 | 0.771 | 0.785 | 0.806 | 0.673 |
| *p-value* | 0.763 | <0.001 | 0.024 | <0.001 | <0.001 | <0.001 | <0.001 | <0.001 | <0.001 | 0.007 | <0.001 | <0.001 | <0.001 | <0.001 | <0.001 | <0.001 |
| **GM-CSF** | 0.538 | 0.413 | 0.297 | 0.478 | 0.422 | 0.357 | 0.573 | 0.387 | 0.465 | 0.478 | 0.53 | 0.464 | 0.376 | 0.42 | 0.412 | 0.424 |
| *p-value* | <0.001 | <0.001 | <0.001 | <0.001 | <0.001 | <0.001 | <0.001 | <0.001 | <0.001 | 0.001 | <0.001 | <0.001 | <0.001 | <0.001 | <0.001 | <0.001 |
| **GRO-alpha** | 0.167 | 0.273 | 0.011 | 0.354 | 0.745 | 0.303 | 0.131 | 0.416 | -0.017 | 0.624 | 0.48 | 0.415 | 0.459 | 0.539 | 0.515 | 0.348 |
| *p-value* | 0.231 | 0.003 | 0.891 | <0.001 | <0.001 | <0.001 | 0.113 | <0.001 | 0.832 | <0.001 | <0.001 | <0.001 | <0.001 | <0.001 | <0.001 | <0.001 |
| **HGF** | -0.066 | 0.337 | -0.021 | 0.825 | 0.3 | 0.685 | 0.453 | 0.738 | 0.517 | 0.401 | 0.842 | 0.798 | 0.821 | 0.764 | 0.869 | 0.482 |
| *p-value* | 0.615 | <0.001 | 0.732 | <0.001 | <0.001 | <0.001 | <0.001 | <0.001 | <0.001 | 0.003 | <0.001 | <0.001 | <0.001 | <0.001 | <0.001 | <0.001 |
| **IFN-alpha** | 0.361 | 0.385 | 0.321 | 0.597 | 0.617 | 0.458 | 0.45 | 0.558 | 0.337 | 0.498 | 0.626 | 0.599 | 0.573 | 0.664 | 0.582 | 0.678 |
| *p-value* | 0.005 | <0.001 | <0.001 | <0.001 | <0.001 | <0.001 | <0.001 | <0.001 | <0.001 | <0.001 | <0.001 | <0.001 | <0.001 | <0.001 | <0.001 | <0.001 |
| **IFN-gamma** | 0.227 | 0.496 | 0.045 | 0.615 | 0.602 | 0.481 | 0.35 | 0.477 | 0.246 | 0.421 | 0.648 | 0.561 | 0.595 | 0.714 | 0.594 | 0.79 |
| *p-value* | 0.079 | <0.001 | 0.466 | <0.001 | <0.001 | <0.001 | <0.001 | <0.001 | <0.001 | 0.002 | <0.001 | <0.001 | <0.001 | <0.001 | <0.001 | <0.001 |
| **IL-1-alpha** | -0.1 | 0.295 | 0.095 | 0.726 | 0.3 | 0.619 | 0.49 | 0.796 | 0.541 | 0.383 | 0.735 | 0.875 | 0.686 | 0.676 | 0.916 | 0.442 |
| *p-value* | 0.445 | <0.001 | 0.12 | <0.001 | <0.001 | <0.001 | <0.001 | <0.001 | <0.001 | 0.006 | <0.001 | <0.001 | <0.001 | <0.001 | <0.001 | <0.001 |
| **IL-1-beta** | 0.103 | 0.249 | 0.283 | 0.675 | 0.346 | 0.548 | 0.563 | 0.592 | 0.771 | 0.426 | 0.661 | 0.668 | 0.603 | 0.563 | 0.612 | 0.509 |
| *p-value* | 0.429 | 0.001 | <0.001 | <0.001 | <0.001 | <0.001 | <0.001 | <0.001 | <0.001 | 0.002 | <0.001 | <0.001 | <0.001 | <0.001 | <0.001 | <0.001 |
| **IL-1RA** | 0.22 | 0.499 | 0.083 | 0.556 | 0.475 | 0.409 | 0.322 | 0.343 | 0.113 | 0.319 | 0.547 | 0.37 | 0.504 | 0.631 | 0.416 | 0.764 |
| *p-value* | 0.089 | <0.001 | 0.174 | <0.001 | <0.001 | <0.001 | <0.001 | <0.001 | 0.053 | 0.021 | <0.001 | <0.001 | <0.001 | <0.001 | <0.001 | <0.001 |
| **IL-2** | 0.466 | 0.436 | 0.306 | 0.623 | 0.572 | 0.567 | 0.54 | 0.649 | 0.358 | 0.67 | 0.605 | 0.61 | 0.585 | 0.6 | 0.644 | 0.577 |
| *p-value* | <0.001 | <0.001 | <0.001 | <0.001 | <0.001 | <0.001 | <0.001 | <0.001 | <0.001 | <0.001 | <0.001 | <0.001 | <0.001 | <0.001 | <0.001 | <0.001 |
| **IL2-R-alpha** | 0.09 | 0.374 | 0.012 | 0.768 | 0.481 | 0.646 | 0.483 | 0.685 | 0.468 | 0.44 | 0.877 | 0.753 | 0.83 | 0.776 | 0.829 | 0.537 |
| *p-value* | 0.489 | <0.001 | 0.847 | <0.001 | <0.001 | <0.001 | <0.001 | <0.001 | <0.001 | 0.001 | <0.001 | <0.001 | <0.001 | <0.001 | <0.001 | <0.001 |
| **IL-3** | -0.144 | 0.127 | -0.108 | 0.621 | 0.336 | 0.568 | 0.378 | 0.781 | 0.401 | 0.198 | 0.627 | 0.735 | 0.587 | 0.629 | 0.817 | 0.402 |
| *p-value* | 0.318 | 0.181 | 0.193 | <0.001 | <0.001 | <0.001 | <0.001 | <0.001 | <0.001 | 0.21 | <0.001 | <0.001 | <0.001 | <0.001 | <0.001 | <0.001 |
| **IL-4** | 0.541 | 0.451 | 0.553 | 0.696 | 0.559 | 0.586 | 0.583 | 0.673 | 0.489 | 0.569 | 0.658 | 0.724 | 0.627 | 0.627 | 0.636 | 0.614 |
| *p-value* | <0.001 | <0.001 | <0.001 | <0.001 | <0.001 | <0.001 | <0.001 | <0.001 | <0.001 | <0.001 | <0.001 | <0.001 | <0.001 | <0.001 | <0.001 | <0.001 |
| **IL-5** | 1 | 0.589 | 0.482 | 0.16 | 0.35 | 0.069 | 0.381 | 0.106 | 0.047 | 0.531 | 0.204 | 0.107 | 0.07 | 0.238 | 0.03 | 0.318 |
| *p-value* | <0.001 | <0.001 | <0.001 | 0.219 | 0.006 | 0.598 | 0.005 | 0.428 | 0.724 | 0.001 | 0.115 | 0.411 | 0.592 | 0.067 | 0.817 | 0.012 |
| **IL-6** | 0.589 | 1 | 0.304 | 0.459 | 0.297 | 0.373 | 0.454 | 0.365 | 0.143 | 0.731 | 0.428 | 0.341 | 0.346 | 0.398 | 0.309 | 0.56 |
| *p-value* | <0.001 | <0.001 | <0.001 | <0.001 | <0.001 | <0.001 | <0.001 | <0.001 | 0.065 | <0.001 | <0.001 | <0.001 | <0.001 | <0.001 | <0.001 | <0.001 |
| **IL-7** | 0.482 | 0.304 | 1 | 0.198 | 0.144 | 0.199 | 0.307 | 0.278 | 0.286 | 0.508 | 0.081 | 0.246 | 0.024 | 0.025 | 0.069 | 0.157 |
| *p-value* | <0.001 | <0.001 | <0.001 | 0.001 | 0.017 | 0.001 | <0.001 | <0.001 | <0.001 | <0.001 | 0.185 | <0.001 | 0.697 | 0.681 | 0.261 | 0.01 |
| **IL-8** | 0.16 | 0.459 | 0.198 | 1 | 0.352 | 0.72 | 0.563 | 0.68 | 0.513 | 0.418 | 0.819 | 0.773 | 0.764 | 0.748 | 0.781 | 0.634 |
| *p-value* | 0.219 | <0.001 | 0.001 | <0.001 | <0.001 | <0.001 | <0.001 | <0.001 | <0.001 | 0.002 | <0.001 | <0.001 | <0.001 | <0.001 | <0.001 | <0.001 |
| **IL-9** | 0.35 | 0.297 | 0.144 | 0.352 | 1 | 0.237 | 0.27 | 0.373 | 0.138 | 0.488 | 0.477 | 0.381 | 0.45 | 0.481 | 0.398 | 0.544 |
| *p-value* | 0.006 | <0.001 | 0.017 | <0.001 | <0.001 | <0.001 | <0.001 | <0.001 | 0.017 | <0.001 | <0.001 | <0.001 | <0.001 | <0.001 | <0.001 | <0.001 |
| **IL-10** | 0.069 | 0.373 | 0.199 | 0.72 | 0.237 | 1 | 0.438 | 0.612 | 0.449 | 0.324 | 0.674 | 0.668 | 0.696 | 0.647 | 0.656 | 0.46 |
| *p-value* | 0.598 | <0.001 | 0.001 | <0.001 | <0.001 | <0.001 | <0.001 | <0.001 | <0.001 | 0.02 | <0.001 | <0.001 | <0.001 | <0.001 | <0.001 | <0.001 |
| **IL-12p70** | 0.381 | 0.454 | 0.307 | 0.563 | 0.27 | 0.438 | 1 | 0.433 | 0.523 | 0.745 | 0.533 | 0.512 | 0.439 | 0.451 | 0.498 | 0.436 |
| *p-value* | 0.005 | <0.001 | <0.001 | <0.001 | <0.001 | <0.001 | <0.001 | <0.001 | <0.001 | <0.001 | <0.001 | <0.001 | <0.001 | <0.001 | <0.001 | <0.001 |
| **IL-12p40** | 0.106 | 0.365 | 0.278 | 0.68 | 0.373 | 0.612 | 0.433 | 1 | 0.499 | 0.355 | 0.633 | 0.803 | 0.608 | 0.587 | 0.798 | 0.404 |
| *p-value* | 0.428 | <0.001 | <0.001 | <0.001 | <0.001 | <0.001 | <0.001 | <0.001 | <0.001 | 0.013 | <0.001 | <0.001 | <0.001 | <0.001 | <0.001 | <0.001 |
| **IL-13** | 0.047 | 0.143 | 0.286 | 0.513 | 0.138 | 0.449 | 0.523 | 0.499 | 1 | 0.246 | 0.426 | 0.571 | 0.385 | 0.305 | 0.493 | 0.279 |
| *p-value* | 0.724 | 0.065 | <0.001 | <0.001 | 0.017 | <0.001 | <0.001 | <0.001 | <0.001 | 0.086 | <0.001 | <0.001 | <0.001 | <0.001 | <0.001 | <0.001 |
| **IL-15** | 0.531 | 0.731 | 0.508 | 0.418 | 0.488 | 0.324 | 0.745 | 0.355 | 0.246 | 1 | 0.582 | 0.431 | 0.341 | 0.501 | 0.408 | 0.415 |
| *p-value* | 0.001 | <0.001 | <0.001 | 0.002 | <0.001 | 0.02 | <0.001 | 0.013 | 0.086 | <0.001 | <0.001 | 0.001 | 0.013 | <0.001 | 0.003 | 0.002 |
| **IL-16** | 0.204 | 0.428 | 0.081 | 0.819 | 0.477 | 0.674 | 0.533 | 0.633 | 0.426 | 0.582 | 1 | 0.749 | 0.858 | 0.838 | 0.818 | 0.616 |
| *p-value* | 0.115 | <0.001 | 0.185 | <0.001 | <0.001 | <0.001 | <0.001 | <0.001 | <0.001 | <0.001 | <0.001 | <0.001 | <0.001 | <0.001 | <0.001 | <0.001 |
| **IL-17** | 0.107 | 0.341 | 0.246 | 0.773 | 0.381 | 0.668 | 0.512 | 0.803 | 0.571 | 0.431 | 0.749 | 1 | 0.694 | 0.702 | 0.864 | 0.536 |
| *p-value* | 0.411 | <0.001 | <0.001 | <0.001 | <0.001 | <0.001 | <0.001 | <0.001 | <0.001 | 0.001 | <0.001 | <0.001 | <0.001 | <0.001 | <0.001 | <0.001 |
| **IL-18** | 0.07 | 0.346 | 0.024 | 0.764 | 0.45 | 0.696 | 0.439 | 0.608 | 0.385 | 0.341 | 0.858 | 0.694 | 1 | 0.781 | 0.777 | 0.581 |
| *p-value* | 0.592 | <0.001 | 0.697 | <0.001 | <0.001 | <0.001 | <0.001 | <0.001 | <0.001 | 0.013 | <0.001 | <0.001 | <0.001 | <0.001 | <0.001 | <0.001 |
| **IP10** | 0.238 | 0.398 | 0.025 | 0.748 | 0.481 | 0.647 | 0.451 | 0.587 | 0.305 | 0.501 | 0.838 | 0.702 | 0.781 | 1 | 0.789 | 0.73 |
| *p-value* | 0.067 | <0.001 | 0.681 | <0.001 | <0.001 | <0.001 | <0.001 | <0.001 | <0.001 | <0.001 | <0.001 | <0.001 | <0.001 | <0.001 | <0.001 | <0.001 |
| **LIF** | 0.03 | 0.309 | 0.069 | 0.781 | 0.398 | 0.656 | 0.498 | 0.798 | 0.493 | 0.408 | 0.818 | 0.864 | 0.777 | 0.789 | 1 | 0.518 |
| *p-value* | 0.817 | <0.001 | 0.261 | <0.001 | <0.001 | <0.001 | <0.001 | <0.001 | <0.001 | 0.003 | <0.001 | <0.001 | <0.001 | <0.001 | <0.001 | <0.001 |
| **MCP1/MCAF** | 0.318 | 0.56 | 0.157 | 0.634 | 0.544 | 0.46 | 0.436 | 0.404 | 0.279 | 0.415 | 0.616 | 0.536 | 0.581 | 0.73 | 0.518 | 1 |
| *p-value* | 0.012 | <0.001 | 0.01 | <0.001 | <0.001 | <0.001 | <0.001 | <0.001 | <0.001 | 0.002 | <0.001 | <0.001 | <0.001 | <0.001 | <0.001 | <0.001 |
| **MCP3** | 0.232 | 0.466 | 0.153 | 0.797 | 0.537 | 0.659 | 0.441 | 0.691 | 0.35 | 0.426 | 0.794 | 0.751 | 0.744 | 0.851 | 0.79 | 0.755 |
| *p-value* | 0.073 | <0.001 | 0.013 | <0.001 | <0.001 | <0.001 | <0.001 | <0.001 | <0.001 | 0.002 | <0.001 | <0.001 | <0.001 | <0.001 | <0.001 | <0.001 |
| **M-CSF** | 0.176 | 0.492 | 0.01 | 0.765 | 0.47 | 0.651 | 0.51 | 0.625 | 0.405 | 0.576 | 0.883 | 0.7 | 0.874 | 0.828 | 0.786 | 0.679 |
| *p-value* | 0.176 | <0.001 | 0.867 | <0.001 | <0.001 | <0.001 | <0.001 | <0.001 | <0.001 | <0.001 | <0.001 | <0.001 | <0.001 | <0.001 | <0.001 | <0.001 |
| **MIF** | 0.177 | 0.37 | 0.087 | 0.7 | 0.504 | 0.579 | 0.447 | 0.552 | 0.422 | 0.241 | 0.742 | 0.605 | 0.711 | 0.674 | 0.616 | 0.56 |
| *p-value* | 0.172 | <0.001 | 0.157 | <0.001 | <0.001 | <0.001 | <0.001 | <0.001 | <0.001 | 0.086 | <0.001 | <0.001 | <0.001 | <0.001 | <0.001 | <0.001 |
| **MIG** | 0.077 | 0.355 | -0.043 | 0.752 | 0.392 | 0.656 | 0.365 | 0.668 | 0.353 | 0.334 | 0.818 | 0.778 | 0.777 | 0.84 | 0.843 | 0.547 |
| *p-value* | 0.553 | <0.001 | 0.483 | <0.001 | <0.001 | <0.001 | <0.001 | <0.001 | <0.001 | 0.016 | <0.001 | <0.001 | <0.001 | <0.001 | <0.001 | <0.001 |
| **MIP1-alpha** | 0.052 | 0.371 | 0.117 | 0.867 | 0.365 | 0.748 | 0.513 | 0.661 | 0.478 | 0.384 | 0.861 | 0.82 | 0.794 | 0.78 | 0.818 | 0.637 |
| *p-value* | 0.692 | <0.001 | 0.054 | <0.001 | <0.001 | <0.001 | <0.001 | <0.001 | <0.001 | 0.005 | <0.001 | <0.001 | <0.001 | <0.001 | <0.001 | <0.001 |
| **MIP1-beta** | 0.428 | 0.474 | 0.176 | 0.538 | 0.933 | 0.373 | 0.397 | 0.439 | 0.226 | 0.595 | 0.622 | 0.488 | 0.592 | 0.615 | 0.507 | 0.685 |
| *p-value* | 0.001 | <0.001 | 0.004 | <0.001 | <0.001 | <0.001 | <0.001 | <0.001 | <0.001 | <0.001 | <0.001 | <0.001 | <0.001 | <0.001 | <0.001 | <0.001 |
| **Beta-NGF** | 0.22 | 0.353 | -0.094 | 0.695 | 0.69 | 0.511 | 0.474 | 0.647 | 0.329 | 0.589 | 0.74 | 0.724 | 0.688 | 0.68 | 0.796 | 0.484 |
| *p-value* | 0.104 | <0.001 | 0.261 | <0.001 | <0.001 | <0.001 | <0.001 | <0.001 | <0.001 | <0.001 | <0.001 | <0.001 | <0.001 | <0.001 | <0.001 | <0.001 |
| **PDGF-BB** | 0.091 | 0.188 | -0.053 | 0.439 | 0.525 | 0.401 | 0.162 | 0.484 | -0.01 | 0.17 | 0.579 | 0.546 | 0.558 | 0.662 | 0.664 | 0.354 |
| *p-value* | 0.488 | 0.019 | 0.438 | <0.001 | <0.001 | <0.001 | 0.017 | <0.001 | 0.88 | 0.228 | <0.001 | <0.001 | <0.001 | <0.001 | <0.001 | <0.001 |
| **RANTES** | 0.147 | 0.26 | 0.007 | 0.362 | 0.926 | 0.223 | 0.237 | 0.379 | 0.116 | 0.224 | 0.5 | 0.378 | 0.461 | 0.503 | 0.453 | 0.521 |
| *p-value* | 0.288 | 0.001 | 0.909 | <0.001 | <0.001 | <0.001 | <0.001 | <0.001 | 0.05 | 0.164 | <0.001 | <0.001 | <0.001 | <0.001 | <0.001 | <0.001 |
| **SCF** | 0.048 | 0.406 | 0.05 | 0.784 | 0.491 | 0.641 | 0.535 | 0.695 | 0.479 | 0.571 | 0.878 | 0.776 | 0.815 | 0.79 | 0.837 | 0.627 |
| *p-value* | 0.713 | <0.001 | 0.415 | <0.001 | <0.001 | <0.001 | <0.001 | <0.001 | <0.001 | <0.001 | <0.001 | <0.001 | <0.001 | <0.001 | <0.001 | <0.001 |
| **SCGF-beta** | -0.108 | 0.181 | -0.04 | 0.744 | 0.226 | 0.626 | 0.439 | 0.634 | 0.511 | 0.375 | 0.783 | 0.75 | 0.774 | 0.739 | 0.858 | 0.411 |
| *p-value* | 0.408 | 0.019 | 0.512 | <0.001 | <0.001 | <0.001 | <0.001 | <0.001 | <0.001 | 0.006 | <0.001 | <0.001 | <0.001 | <0.001 | <0.001 | <0.001 |
| **SDF-1-alpha** | 0.319 | 0.378 | 0.02 | 0.673 | 0.626 | 0.568 | 0.359 | 0.579 | 0.261 | 0.555 | 0.78 | 0.677 | 0.765 | 0.837 | 0.746 | 0.64 |
| *p-value* | 0.012 | <0.001 | 0.743 | <0.001 | <0.001 | <0.001 | <0.001 | <0.001 | <0.001 | <0.001 | <0.001 | <0.001 | <0.001 | <0.001 | <0.001 | <0.001 |
| **TNF-alpha** | 0.087 | 0.346 | 0.156 | 0.735 | 0.57 | 0.614 | 0.488 | 0.733 | 0.491 | 0.394 | 0.784 | 0.835 | 0.713 | 0.717 | 0.838 | 0.578 |
| *p-value* | 0.504 | <0.001 | 0.01 | <0.001 | <0.001 | <0.001 | <0.001 | <0.001 | <0.001 | 0.004 | <0.001 | <0.001 | <0.001 | <0.001 | <0.001 | <0.001 |
| **TNF-beta** | 0.379 | 0.342 | 0.175 | 0.359 | 0.98 | 0.213 | 0.308 | 0.352 | 0.153 | 0.482 | 0.468 | 0.359 | 0.439 | 0.45 | 0.375 | 0.542 |
| *p-value* | 0.003 | <0.001 | 0.004 | <0.001 | <0.001 | <0.001 | <0.001 | <0.001 | 0.008 | <0.001 | <0.001 | <0.001 | <0.001 | <0.001 | <0.001 | <0.001 |
| **TRAIL** | -0.172 | 0.257 | -0.126 | 0.702 | 0.494 | 0.539 | 0.349 | 0.637 | 0.361 | 0.327 | 0.795 | 0.736 | 0.75 | 0.808 | 0.868 | 0.607 |
| *p-value* | 0.186 | 0.001 | 0.037 | <0.001 | <0.001 | <0.001 | <0.001 | <0.001 | <0.001 | 0.018 | <0.001 | <0.001 | <0.001 | <0.001 | <0.001 | <0.001 |
| **VEGF** | 0.499 | 0.282 | 0.243 | 0.257 | 0.553 | 0.147 | 0.226 | 0.195 | -0.005 | 0.554 | 0.341 | 0.227 | 0.252 | 0.357 | 0.308 | 0.241 |
| *p-value* | <0.001 | 0.007 | 0.02 | 0.01 | <0.001 | 0.151 | 0.029 | 0.057 | 0.959 | <0.001 | 0.001 | 0.024 | 0.012 | <0.001 | 0.002 | 0.016 |

|  | **MCP3** | **M-CSF** | **MIF** | **MIG** | **MIP1-alpha** | **MIP1-beta** | **Beta-NGF** | **PDGF-BB** | **RANTES** | **SCF** | **SCGF-beta** | **SDF-1-alpha** | **TNF-alpha** | **TNF-beta** | **TRAIL** | **VEGF** |
| --- | --- | --- | --- | --- | --- | --- | --- | --- | --- | --- | --- | --- | --- | --- | --- | --- |
| **CTACK** | 0.745 | 0.774 | 0.637 | 0.882 | 0.802 | 0.491 | 0.776 | 0.673 | 0.452 | 0.843 | 0.882 | 0.821 | 0.775 | 0.343 | 0.839 | 0.236 |
| *p-value* | <0.001 | <0.001 | <0.001 | <0.001 | <0.001 | <0.001 | <0.001 | <0.001 | <0.001 | <0.001 | <0.001 | <0.001 | <0.001 | <0.001 | <0.001 | 0.019 |
| **Eotaxin** | 0.779 | 0.705 | 0.67 | 0.78 | 0.724 | 0.478 | 0.665 | 0.538 | 0.385 | 0.756 | 0.778 | 0.743 | 0.708 | 0.325 | 0.764 | 0.157 |
| *p-value* | <0.001 | <0.001 | <0.001 | <0.001 | <0.001 | <0.001 | <0.001 | <0.001 | <0.001 | <0.001 | <0.001 | <0.001 | <0.001 | <0.001 | <0.001 | 0.121 |
| **FGF-basic** | 0.785 | 0.766 | 0.56 | 0.871 | 0.804 | 0.523 | 0.837 | 0.729 | 0.48 | 0.839 | 0.825 | 0.828 | 0.832 | 0.368 | 0.909 | 0.307 |
| *p-value* | <0.001 | <0.001 | <0.001 | <0.001 | <0.001 | <0.001 | <0.001 | <0.001 | <0.001 | <0.001 | <0.001 | <0.001 | <0.001 | <0.001 | <0.001 | 0.002 |
| **G-CSF** | 0.863 | 0.786 | 0.67 | 0.779 | 0.913 | 0.629 | 0.723 | 0.565 | 0.495 | 0.797 | 0.675 | 0.742 | 0.816 | 0.466 | 0.777 | 0.343 |
| *p-value* | <0.001 | <0.001 | <0.001 | <0.001 | <0.001 | <0.001 | <0.001 | <0.001 | <0.001 | <0.001 | <0.001 | <0.001 | <0.001 | <0.001 | <0.001 | 0.001 |
| **GM-CSF** | 0.417 | 0.42 | 0.427 | 0.381 | 0.43 | 0.488 | 0.423 | 0.215 | 0.355 | 0.428 | 0.318 | 0.371 | 0.474 | 0.452 | 0.292 | 0.318 |
| *p-value* | <0.001 | <0.001 | <0.001 | <0.001 | <0.001 | <0.001 | <0.001 | 0.003 | <0.001 | <0.001 | <0.001 | <0.001 | <0.001 | <0.001 | <0.001 | 0.002 |
| **GRO-alpha** | 0.637 | 0.497 | 0.318 | 0.536 | 0.394 | 0.68 | 0.789 | 0.689 | 0.663 | 0.586 | 0.272 | 0.822 | 0.617 | 0.657 | 0.619 | 0.577 |
| *p-value* | <0.001 | <0.001 | <0.001 | <0.001 | <0.001 | <0.001 | <0.001 | <0.001 | <0.001 | <0.001 | 0.001 | <0.001 | <0.001 | <0.001 | <0.001 | <0.001 |
| **HGF** | 0.734 | 0.811 | 0.688 | 0.846 | 0.827 | 0.448 | 0.747 | 0.546 | 0.369 | 0.847 | 0.898 | 0.723 | 0.757 | 0.285 | 0.811 | 0.152 |
| *p-value* | <0.001 | <0.001 | <0.001 | <0.001 | <0.001 | <0.001 | <0.001 | <0.001 | <0.001 | <0.001 | <0.001 | <0.001 | <0.001 | <0.001 | <0.001 | 0.133 |
| **IFN-alpha** | 0.742 | 0.637 | 0.599 | 0.478 | 0.541 | 0.688 | 0.541 | 0.468 | 0.556 | 0.622 | 0.41 | 0.636 | 0.628 | 0.597 | 0.57 | 0.35 |
| *p-value* | <0.001 | <0.001 | <0.001 | <0.001 | <0.001 | <0.001 | <0.001 | <0.001 | <0.001 | <0.001 | <0.001 | <0.001 | <0.001 | <0.001 | <0.001 | 0.001 |
| **IFN-gamma** | 0.804 | 0.737 | 0.578 | 0.611 | 0.624 | 0.694 | 0.566 | 0.467 | 0.577 | 0.664 | 0.417 | 0.65 | 0.632 | 0.576 | 0.706 | 0.34 |
| *p-value* | <0.001 | <0.001 | <0.001 | <0.001 | <0.001 | <0.001 | <0.001 | <0.001 | <0.001 | <0.001 | <0.001 | <0.001 | <0.001 | <0.001 | <0.001 | 0.001 |
| **IL-1-alpha** | 0.736 | 0.698 | 0.548 | 0.774 | 0.79 | 0.403 | 0.699 | 0.569 | 0.367 | 0.786 | 0.787 | 0.627 | 0.827 | 0.276 | 0.8 | 0.191 |
| *p-value* | <0.001 | <0.001 | <0.001 | <0.001 | <0.001 | <0.001 | <0.001 | <0.001 | <0.001 | <0.001 | <0.001 | <0.001 | <0.001 | <0.001 | <0.001 | 0.059 |
| **IL-1-beta** | 0.609 | 0.617 | 0.639 | 0.528 | 0.643 | 0.459 | 0.474 | 0.173 | 0.322 | 0.662 | 0.609 | 0.493 | 0.652 | 0.359 | 0.535 | 0.159 |
| *p-value* | <0.001 | <0.001 | <0.001 | <0.001 | <0.001 | <0.001 | <0.001 | 0.007 | <0.001 | <0.001 | <0.001 | <0.001 | <0.001 | <0.001 | <0.001 | 0.116 |
| **IL-1RA** | 0.695 | 0.66 | 0.522 | 0.399 | 0.542 | 0.604 | 0.393 | 0.278 | 0.477 | 0.521 | 0.279 | 0.472 | 0.466 | 0.488 | 0.558 | 0.24 |
| *p-value* | <0.001 | <0.001 | <0.001 | <0.001 | <0.001 | <0.001 | <0.001 | <0.001 | <0.001 | <0.001 | <0.001 | <0.001 | <0.001 | <0.001 | <0.001 | 0.017 |
| **IL-2** | 0.665 | 0.591 | 0.485 | 0.568 | 0.571 | 0.618 | 0.715 | 0.426 | 0.463 | 0.628 | 0.489 | 0.608 | 0.607 | 0.548 | 0.471 | 0.632 |
| *p-value* | <0.001 | <0.001 | <0.001 | <0.001 | <0.001 | <0.001 | <0.001 | <0.001 | <0.001 | <0.001 | <0.001 | <0.001 | <0.001 | <0.001 | <0.001 | <0.001 |
| **IL2-R-alpha** | 0.75 | 0.868 | 0.696 | 0.834 | 0.803 | 0.604 | 0.797 | 0.529 | 0.514 | 0.884 | 0.8 | 0.761 | 0.768 | 0.468 | 0.803 | 0.301 |
| *p-value* | <0.001 | <0.001 | <0.001 | <0.001 | <0.001 | <0.001 | <0.001 | <0.001 | <0.001 | <0.001 | <0.001 | <0.001 | <0.001 | <0.001 | <0.001 | 0.002 |
| **IL-3** | 0.673 | 0.606 | 0.56 | 0.723 | 0.661 | 0.36 | 0.609 | 0.464 | 0.405 | 0.679 | 0.648 | 0.577 | 0.734 | 0.3 | 0.713 | 0.205 |
| *p-value* | <0.001 | <0.001 | <0.001 | <0.001 | <0.001 | <0.001 | <0.001 | <0.001 | <0.001 | <0.001 | <0.001 | <0.001 | <0.001 | <0.001 | <0.001 | 0.074 |
| **IL-4** | 0.717 | 0.594 | 0.608 | 0.541 | 0.637 | 0.652 | 0.532 | 0.44 | 0.436 | 0.659 | 0.5 | 0.641 | 0.665 | 0.552 | 0.483 | 0.512 |
| *p-value* | <0.001 | <0.001 | <0.001 | <0.001 | <0.001 | <0.001 | <0.001 | <0.001 | <0.001 | <0.001 | <0.001 | <0.001 | <0.001 | <0.001 | <0.001 | <0.001 |
| **IL-5** | 0.232 | 0.176 | 0.177 | 0.077 | 0.052 | 0.428 | 0.22 | 0.091 | 0.147 | 0.048 | -0.108 | 0.319 | 0.087 | 0.379 | -0.172 | 0.499 |
| *p-value* | 0.073 | 0.176 | 0.172 | 0.553 | 0.692 | 0.001 | 0.104 | 0.488 | 0.288 | 0.713 | 0.408 | 0.012 | 0.504 | 0.003 | 0.186 | <0.001 |
| **IL-6** | 0.466 | 0.492 | 0.37 | 0.355 | 0.371 | 0.474 | 0.353 | 0.188 | 0.26 | 0.406 | 0.181 | 0.378 | 0.346 | 0.342 | 0.257 | 0.282 |
| *p-value* | <0.001 | <0.001 | <0.001 | <0.001 | <0.001 | <0.001 | <0.001 | 0.019 | 0.001 | <0.001 | 0.019 | <0.001 | <0.001 | <0.001 | 0.001 | 0.007 |
| **IL-7** | 0.153 | 0.01 | 0.087 | -0.043 | 0.117 | 0.176 | -0.094 | -0.053 | 0.007 | 0.05 | -0.04 | 0.02 | 0.156 | 0.175 | -0.126 | 0.243 |
| *p-value* | 0.013 | 0.867 | 0.157 | 0.483 | 0.054 | 0.004 | 0.261 | 0.438 | 0.909 | 0.415 | 0.512 | 0.743 | 0.01 | 0.004 | 0.037 | 0.02 |
| **IL-8** | 0.797 | 0.765 | 0.7 | 0.752 | 0.867 | 0.538 | 0.695 | 0.439 | 0.362 | 0.784 | 0.744 | 0.673 | 0.735 | 0.359 | 0.702 | 0.257 |
| *p-value* | <0.001 | <0.001 | <0.001 | <0.001 | <0.001 | <0.001 | <0.001 | <0.001 | <0.001 | <0.001 | <0.001 | <0.001 | <0.001 | <0.001 | <0.001 | 0.01 |
| **IL-9** | 0.537 | 0.47 | 0.504 | 0.392 | 0.365 | 0.933 | 0.69 | 0.525 | 0.926 | 0.491 | 0.226 | 0.626 | 0.57 | 0.98 | 0.494 | 0.553 |
| *p-value* | <0.001 | <0.001 | <0.001 | <0.001 | <0.001 | <0.001 | <0.001 | <0.001 | <0.001 | <0.001 | <0.001 | <0.001 | <0.001 | <0.001 | <0.001 | <0.001 |
| **IL-10** | 0.659 | 0.651 | 0.579 | 0.656 | 0.748 | 0.373 | 0.511 | 0.401 | 0.223 | 0.641 | 0.626 | 0.568 | 0.614 | 0.213 | 0.539 | 0.147 |
| *p-value* | <0.001 | <0.001 | <0.001 | <0.001 | <0.001 | <0.001 | <0.001 | <0.001 | <0.001 | <0.001 | <0.001 | <0.001 | <0.001 | <0.001 | <0.001 | 0.151 |
| **IL-12p70** | 0.441 | 0.51 | 0.447 | 0.365 | 0.513 | 0.397 | 0.474 | 0.162 | 0.237 | 0.535 | 0.439 | 0.359 | 0.488 | 0.308 | 0.349 | 0.226 |
| *p-value* | <0.001 | <0.001 | <0.001 | <0.001 | <0.001 | <0.001 | <0.001 | 0.017 | <0.001 | <0.001 | <0.001 | <0.001 | <0.001 | <0.001 | <0.001 | 0.029 |
| **IL-12p40** | 0.691 | 0.625 | 0.552 | 0.668 | 0.661 | 0.439 | 0.647 | 0.484 | 0.379 | 0.695 | 0.634 | 0.579 | 0.733 | 0.352 | 0.637 | 0.195 |
| *p-value* | <0.001 | <0.001 | <0.001 | <0.001 | <0.001 | <0.001 | <0.001 | <0.001 | <0.001 | <0.001 | <0.001 | <0.001 | <0.001 | <0.001 | <0.001 | 0.057 |
| **IL-13** | 0.35 | 0.405 | 0.422 | 0.353 | 0.478 | 0.226 | 0.329 | -0.01 | 0.116 | 0.479 | 0.511 | 0.261 | 0.491 | 0.153 | 0.361 | -0.005 |
| *p-value* | <0.001 | <0.001 | <0.001 | <0.001 | <0.001 | <0.001 | <0.001 | 0.88 | 0.05 | <0.001 | <0.001 | <0.001 | <0.001 | 0.008 | <0.001 | 0.959 |
| **IL-15** | 0.426 | 0.576 | 0.241 | 0.334 | 0.384 | 0.595 | 0.589 | 0.17 | 0.224 | 0.571 | 0.375 | 0.555 | 0.394 | 0.482 | 0.327 | 0.554 |
| *p-value* | 0.002 | <0.001 | 0.086 | 0.016 | 0.005 | <0.001 | <0.001 | 0.228 | 0.164 | <0.001 | 0.006 | <0.001 | 0.004 | <0.001 | 0.018 | <0.001 |
| **IL-16** | 0.794 | 0.883 | 0.742 | 0.818 | 0.861 | 0.622 | 0.74 | 0.579 | 0.5 | 0.878 | 0.783 | 0.78 | 0.784 | 0.468 | 0.795 | 0.341 |
| *p-value* | <0.001 | <0.001 | <0.001 | <0.001 | <0.001 | <0.001 | <0.001 | <0.001 | <0.001 | <0.001 | <0.001 | <0.001 | <0.001 | <0.001 | <0.001 | 0.001 |
| **IL-17** | 0.751 | 0.7 | 0.605 | 0.778 | 0.82 | 0.488 | 0.724 | 0.546 | 0.378 | 0.776 | 0.75 | 0.677 | 0.835 | 0.359 | 0.736 | 0.227 |
| *p-value* | <0.001 | <0.001 | <0.001 | <0.001 | <0.001 | <0.001 | <0.001 | <0.001 | <0.001 | <0.001 | <0.001 | <0.001 | <0.001 | <0.001 | <0.001 | 0.024 |
| **IL-18** | 0.744 | 0.874 | 0.711 | 0.777 | 0.794 | 0.592 | 0.688 | 0.558 | 0.461 | 0.815 | 0.774 | 0.765 | 0.713 | 0.439 | 0.75 | 0.252 |
| *p-value* | <0.001 | <0.001 | <0.001 | <0.001 | <0.001 | <0.001 | <0.001 | <0.001 | <0.001 | <0.001 | <0.001 | <0.001 | <0.001 | <0.001 | <0.001 | 0.012 |
| **IP10** | 0.851 | 0.828 | 0.674 | 0.84 | 0.78 | 0.615 | 0.68 | 0.662 | 0.503 | 0.79 | 0.739 | 0.837 | 0.717 | 0.45 | 0.808 | 0.357 |
| *p-value* | <0.001 | <0.001 | <0.001 | <0.001 | <0.001 | <0.001 | <0.001 | <0.001 | <0.001 | <0.001 | <0.001 | <0.001 | <0.001 | <0.001 | <0.001 | <0.001 |
| **LIF** | 0.79 | 0.786 | 0.616 | 0.843 | 0.818 | 0.507 | 0.796 | 0.664 | 0.453 | 0.837 | 0.858 | 0.746 | 0.838 | 0.375 | 0.868 | 0.308 |
| *p-value* | <0.001 | <0.001 | <0.001 | <0.001 | <0.001 | <0.001 | <0.001 | <0.001 | <0.001 | <0.001 | <0.001 | <0.001 | <0.001 | <0.001 | <0.001 | 0.002 |
| **MCP1/MCAF** | 0.755 | 0.679 | 0.56 | 0.547 | 0.637 | 0.685 | 0.484 | 0.354 | 0.521 | 0.627 | 0.411 | 0.64 | 0.578 | 0.542 | 0.607 | 0.241 |
| *p-value* | <0.001 | <0.001 | <0.001 | <0.001 | <0.001 | <0.001 | <0.001 | <0.001 | <0.001 | <0.001 | <0.001 | <0.001 | <0.001 | <0.001 | <0.001 | 0.016 |
| **MCP3** | 1 | 0.791 | 0.7 | 0.786 | 0.812 | 0.659 | 0.726 | 0.63 | 0.539 | 0.778 | 0.633 | 0.786 | 0.779 | 0.501 | 0.791 | 0.376 |
| *p-value* | <0.001 | <0.001 | <0.001 | <0.001 | <0.001 | <0.001 | <0.001 | <0.001 | <0.001 | <0.001 | <0.001 | <0.001 | <0.001 | <0.001 | <0.001 | <0.001 |
| **M-CSF** | 0.791 | 1 | 0.676 | 0.754 | 0.816 | 0.617 | 0.716 | 0.486 | 0.494 | 0.847 | 0.725 | 0.732 | 0.733 | 0.465 | 0.824 | 0.305 |
| *p-value* | <0.001 | <0.001 | <0.001 | <0.001 | <0.001 | <0.001 | <0.001 | <0.001 | <0.001 | <0.001 | <0.001 | <0.001 | <0.001 | <0.001 | <0.001 | 0.002 |
| **MIF** | 0.7 | 0.676 | 1 | 0.627 | 0.652 | 0.61 | 0.531 | 0.47 | 0.521 | 0.69 | 0.618 | 0.651 | 0.628 | 0.499 | 0.613 | 0.019 |
| *p-value* | <0.001 | <0.001 | <0.001 | <0.001 | <0.001 | <0.001 | <0.001 | <0.001 | <0.001 | <0.001 | <0.001 | <0.001 | <0.001 | <0.001 | <0.001 | 0.855 |
| **MIG** | 0.786 | 0.754 | 0.627 | 1 | 0.795 | 0.524 | 0.766 | 0.635 | 0.45 | 0.787 | 0.801 | 0.812 | 0.765 | 0.363 | 0.803 | 0.274 |
| *p-value* | <0.001 | <0.001 | <0.001 | <0.001 | <0.001 | <0.001 | <0.001 | <0.001 | <0.001 | <0.001 | <0.001 | <0.001 | <0.001 | <0.001 | <0.001 | 0.006 |
| **MIP1-alpha** | 0.812 | 0.816 | 0.652 | 0.795 | 1 | 0.546 | 0.697 | 0.466 | 0.397 | 0.809 | 0.775 | 0.702 | 0.81 | 0.359 | 0.792 | 0.217 |
| *p-value* | <0.001 | <0.001 | <0.001 | <0.001 | <0.001 | <0.001 | <0.001 | <0.001 | <0.001 | <0.001 | <0.001 | <0.001 | <0.001 | <0.001 | <0.001 | 0.031 |
| **MIP1-beta** | 0.659 | 0.617 | 0.61 | 0.524 | 0.546 | 1 | 0.645 | 0.524 | 0.895 | 0.627 | 0.375 | 0.735 | 0.672 | 0.943 | 0.604 | 0.562 |
| *p-value* | <0.001 | <0.001 | <0.001 | <0.001 | <0.001 | <0.001 | <0.001 | <0.001 | <0.001 | <0.001 | <0.001 | <0.001 | <0.001 | <0.001 | <0.001 | <0.001 |
| **Beta-NGF** | 0.726 | 0.716 | 0.531 | 0.766 | 0.697 | 0.645 | 1 | 0.635 | 0.69 | 0.795 | 0.735 | 0.779 | 0.8 | 0.653 | 0.772 | 0.516 |
| *p-value* | <0.001 | <0.001 | <0.001 | <0.001 | <0.001 | <0.001 | <0.001 | <0.001 | <0.001 | <0.001 | <0.001 | <0.001 | <0.001 | <0.001 | <0.001 | <0.001 |
| **PDGF-BB** | 0.63 | 0.486 | 0.47 | 0.635 | 0.466 | 0.524 | 0.635 | 1 | 0.541 | 0.586 | 0.501 | 0.744 | 0.63 | 0.46 | 0.691 | 0.4 |
| *p-value* | <0.001 | <0.001 | <0.001 | <0.001 | <0.001 | <0.001 | <0.001 | <0.001 | <0.001 | <0.001 | <0.001 | <0.001 | <0.001 | <0.001 | <0.001 | <0.001 |
| **RANTES** | 0.539 | 0.494 | 0.521 | 0.45 | 0.397 | 0.895 | 0.69 | 0.541 | 1 | 0.518 | 0.311 | 0.607 | 0.602 | 0.93 | 0.566 | 0.386 |
| *p-value* | <0.001 | <0.001 | <0.001 | <0.001 | <0.001 | <0.001 | <0.001 | <0.001 | <0.001 | <0.001 | <0.001 | <0.001 | <0.001 | <0.001 | <0.001 | <0.001 |
| **SCF** | 0.778 | 0.847 | 0.69 | 0.787 | 0.809 | 0.627 | 0.795 | 0.586 | 0.518 | 1 | 0.795 | 0.797 | 0.811 | 0.478 | 0.832 | 0.331 |
| *p-value* | <0.001 | <0.001 | <0.001 | <0.001 | <0.001 | <0.001 | <0.001 | <0.001 | <0.001 | <0.001 | <0.001 | <0.001 | <0.001 | <0.001 | <0.001 | 0.001 |
| **SCGF-beta** | 0.633 | 0.725 | 0.618 | 0.801 | 0.775 | 0.375 | 0.735 | 0.501 | 0.311 | 0.795 | 1 | 0.697 | 0.701 | 0.218 | 0.788 | 0.173 |
| *p-value* | <0.001 | <0.001 | <0.001 | <0.001 | <0.001 | <0.001 | <0.001 | <0.001 | <0.001 | <0.001 | <0.001 | <0.001 | <0.001 | <0.001 | <0.001 | 0.088 |
| **SDF-1-alpha** | 0.786 | 0.732 | 0.651 | 0.812 | 0.702 | 0.735 | 0.779 | 0.744 | 0.607 | 0.797 | 0.697 | 1 | 0.736 | 0.575 | 0.795 | 0.556 |
| *p-value* | <0.001 | <0.001 | <0.001 | <0.001 | <0.001 | <0.001 | <0.001 | <0.001 | <0.001 | <0.001 | <0.001 | <0.001 | <0.001 | <0.001 | <0.001 | <0.001 |
| **TNF-alpha** | 0.779 | 0.733 | 0.628 | 0.765 | 0.81 | 0.672 | 0.8 | 0.63 | 0.602 | 0.811 | 0.701 | 0.736 | 1 | 0.559 | 0.81 | 0.316 |
| *p-value* | <0.001 | <0.001 | <0.001 | <0.001 | <0.001 | <0.001 | <0.001 | <0.001 | <0.001 | <0.001 | <0.001 | <0.001 | <0.001 | <0.001 | <0.001 | 0.001 |
| **TNF-beta** | 0.501 | 0.465 | 0.499 | 0.363 | 0.359 | 0.943 | 0.653 | 0.46 | 0.93 | 0.478 | 0.218 | 0.575 | 0.559 | 1 | 0.464 | 0.536 |
| *p-value* | <0.001 | <0.001 | <0.001 | <0.001 | <0.001 | <0.001 | <0.001 | <0.001 | <0.001 | <0.001 | <0.001 | <0.001 | <0.001 | <0.001 | <0.001 | <0.001 |
| **TRAIL** | 0.791 | 0.824 | 0.613 | 0.803 | 0.792 | 0.604 | 0.772 | 0.691 | 0.566 | 0.832 | 0.788 | 0.795 | 0.81 | 0.464 | 1 | 0.258 |
| *p-value* | <0.001 | <0.001 | <0.001 | <0.001 | <0.001 | <0.001 | <0.001 | <0.001 | <0.001 | <0.001 | <0.001 | <0.001 | <0.001 | <0.001 | <0.001 | 0.01 |
| **VEGF** | 0.376 | 0.305 | 0.019 | 0.274 | 0.217 | 0.562 | 0.516 | 0.4 | 0.386 | 0.331 | 0.173 | 0.556 | 0.316 | 0.536 | 0.258 | 1 |
| *p-value* | <0.001 | 0.002 | 0.855 | 0.006 | 0.031 | <0.001 | <0.001 | <0.001 | <0.001 | 0.001 | 0.088 | <0.001 | 0.001 | <0.001 | 0.01 | <0.001 |

The names of inflammatory mediators are detailed in the abbreviation list. The correlation coefficient was calculated using Spearman's method with Holm corrections. P-value <0.05 is considered significant.

# **Supplementary Figure S1. Feature importance plot and AUROC curve of Model with the most robust predictors**


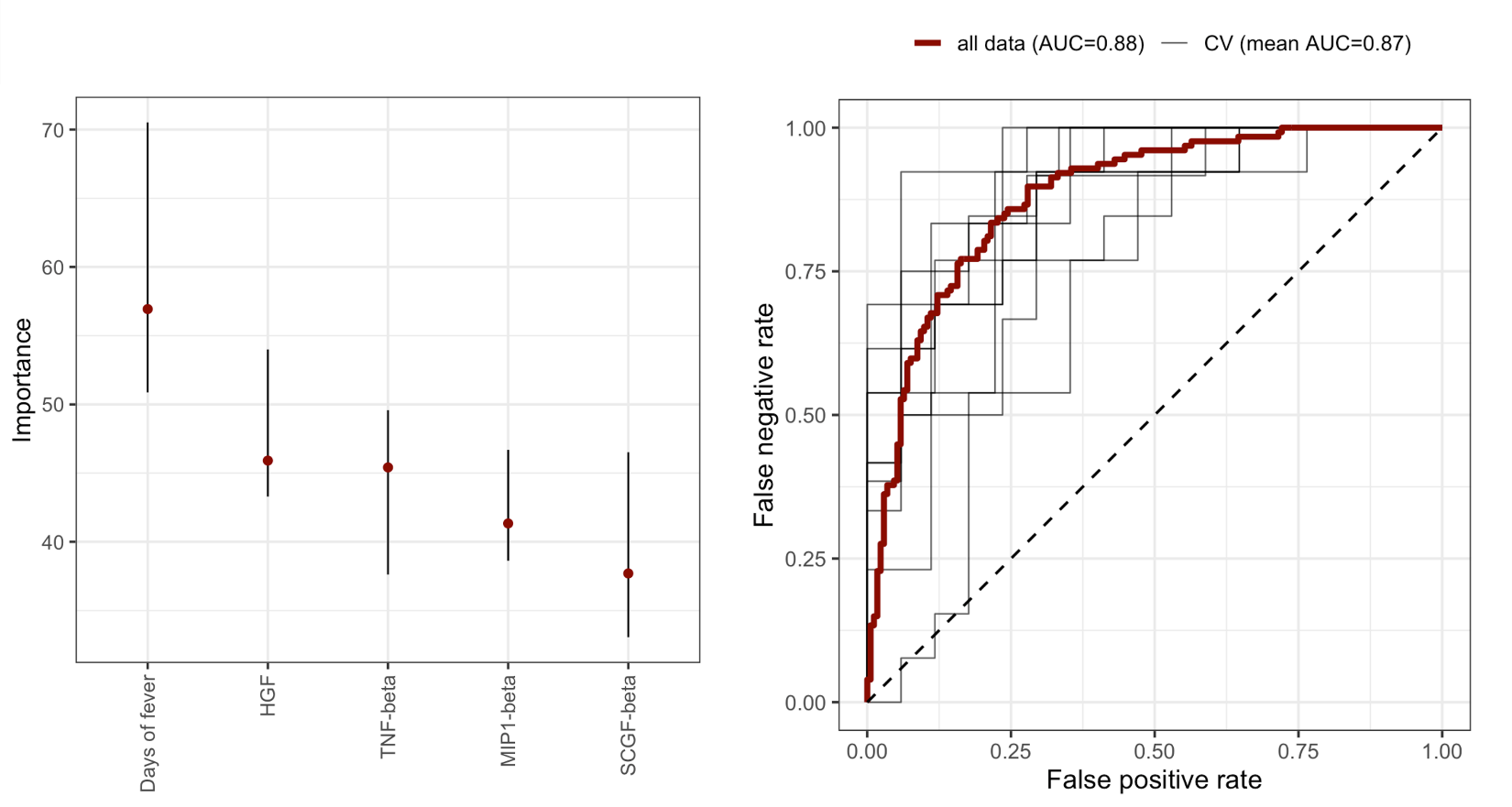

Supplement: Supplementary file 1 — Supplementary Table S1. Plasma levels of inflammatory mediators in dengue patients and healthy controls. Supplementary Table S2. Correlation matrix of plasma levels of inflammatory mediators and laboratory parameters in dengue patients. Supplementary Table S3. Correlation matrix of plasma levels of inflammatory mediators in dengue patients. Supplementary Figure S1. Feature importance plot and AUROC curve of Model with the most robust predictors. [file JMV-97-e70511-s001.docx]
